# Highly-resolved interannual phytoplankton community dynamics of the coastal Northwest Atlantic

**DOI:** 10.1038/s43705-022-00119-2

**Published:** 2022-04-20

**Authors:** Brent M. Robicheau, Jennifer Tolman, Erin M. Bertrand, Julie LaRoche

**Affiliations:** Department of Biology, Dalhousie University, Halifax, NS Canada

**Keywords:** Water microbiology, Microbial ecology, Microbial ecology, Biodiversity

## Abstract

Microbial observatories can track phytoplankton at frequencies that resolve monthly, seasonal, and multiyear trends in environmental change from short-lived events. Using 4-years of weekly flow cytometry along with chloroplast and cyanobacterial *16S* rRNA gene sequence data from a time-series station in the coastal Northwest Atlantic (Bedford Basin, Nova Scotia, Canada), we analyzed temporal observations for globally-relevant genera (e.g., *Bolidomonas*, *Teleaulax*, *Minidiscus*, *Chaetoceros*, *Synechococcus*, and *Phaeocystis*) in an oceanic region that has been recognized as a likely hotspot for phytoplankton diversity. Contemporaneous Scotian Shelf data also collected during our study established that the major phytoplankton within the Bedford Basin were important in the Scotian Shelf during spring and fall, therefore pointing to their broader significance within the coastal Northwest Atlantic (NWA). Temporal trends revealed a subset of indicator taxa along with their DNA signatures (e.g., *Eutreptiella* and *Synechococcus*), whose distribution patterns make them essential for timely detection of environmentally-driven shifts in the NWA. High-resolution sampling was key to identifying important community shifts towards smaller phytoplankton under anomalous environmental conditions, while further providing a detailed molecular view of community compositions underpinning general phytoplankton succession within the coastal NWA. Our study demonstrates the importance of accessible coastal time-series sites where high-frequency DNA sampling allows for the detection of shifting baselines in phytoplankton communities.

## Introduction

Marine phytoplankton contribute ~40% of global carbon fixation and their impact for higher trophic levels, biological carbon uptake, and hence climate, is well recognized [1–3]. In the Northwest Atlantic (NWA) primary production is characterized by spring and fall phytoplankton blooms [4], and sampling at coastal time-series stations has demonstrated a pattern of few larger phytoplankton species (often diatoms) dominating the spring bloom with progressive shifts to higher cell density of smaller phytoplankton species as temperature increases throughout the summer months [5]. However, based on early winter and spring latitudinal transects in the central NWA, Bolaños et al. [6] recently challenged the broadly accepted view that larger diatoms dominate the spring bloom, proposing instead that small phytoplankton species are important members of spring blooms within the North Atlantic.

Long-term microbial observatories are vital for tracking marine microbes [7] and are an important counterpart to the remote sensing of phytoplankton [8]. The Bedford Basin Monitoring Program (BBMP), located in Halifax Harbour, Nova Scotia, Canada [9], represents one of >70 microbial observatories that exist globally [7] (see [10–13] for other examples). Bedford Basin (71 m deep, 8 km long) is connected to the Scotian Shelf [14] and displays characteristic nutrient and phytoplankton annual cycling for the temperate NWA, including annual spring and fall blooms separated by strong summer stratification [5, 9]. As a fjord with a long narrow entrance [15], Bedford Basin experiences limited freshwater input [14, 16] with an approximately three-month flushing time and a net outward flow for surface waters [16, 17]. On a global scale, the NWA coastal waters near the BBMP, in connection to the Gulf Stream, have also been predicted as a hotspot for phytoplankton richness [18–20], and in addition, this region is of special interest because higher latitudes/temperate waters are likely to display a higher turnover of species due to high monthly variability in environmental conditions [21]. The phytoplankton community of the Bedford Basin has been documented using flow cytometry at a basic level since the 1990s [9, 22–24], however, molecular work has been limited and has focused mainly on seasonal non-photosynthetic bacteria, as well as a subset of metaproteomes [25, 26]. Previous studies reported increased diatom cell density in spring phytoplankton blooms in the fjord [5, 27, 28]; while flow cytometry has shown that *Synechococcus* growth coincides with increases in chlorophyll *a* (chl *a*) concentrations in the late summer and fall [5].

Here we present 4-years of weekly sampling in the Bedford Basin that characterizes the phytoplankton communities using chloroplast and cyanobacterial *16S* rRNA gene metabarcodes paired with phytoplankton cell concentrations obtained by flow cytometry. Using these data, we compare the latest cell concentrations and phytoplankton community characteristics to historical records and report on novel phytoplankton diversity trends observed within this coastal system. Indicator species—organisms associated with a specific set of environmental conditions [29]—were also identified and may be important in tracking environmental changes in the NWA in general. Using a comparable metabarcoding dataset from a transect across the Scotian Shelf towards the Gulf Stream, we also show that >80% of the major phytoplankton identified in the Bedford Basin time series were also present in phytoplankton communities of the Scotian Shelf during spring and fall, and that there was a general overlap in the dominant phytoplankton present at both the Scotian Shelf and inshore basin. Finally, we use our high-resolution multi-parameter sampling to highlight atypical phytoplankton community shifts that occurred in relation to anomalous nutrient and temperature events.

## Materials and methods

### Sampling, oceanographic data, and flow cytometry

Water samples were collected using Niskin bottles from 1, 5, 10, and 60 m depths weekly from Bedford Basin (BB; 44.6936, −63.6403; Halifax, Nova Scotia, Canada), and transported in dark bottles kept in a cooler to a laboratory at Dalhousie University (NS, Canada) and processed immediately upon arrival.

For flow cytometry, 2.5 mL of seawater per depth was prefiltered using 35μm cell strainers and autofluorescent cell counts were recorded on a CSampler-equipped BD Accuri^TM^ C6 Flow Cytometer (BD Biosciences, USA) with optical filters for Chlorophyll [>670 nm] and Phycoerythrin [585/40 nm] detection. The flow cytometry approach implemented targeted ~1–35μm cells, and cell counts were corrected using blanks (0.2μm-filtered seawater) measured concurrently each week (see Supplementary Methods S1 for cytometry gate details, and Supplementary Data S1 for count data).

DNA samples were filtered using acid-washed tubing and bottles and a peristaltic pump. Seawater (500 mL) was prefiltered using mesh (at 160μm in 2014–2015, and at 330μm in 2016–2017) and then filtered onto 0.2μm polycarbonate Isopore filters (Millipore, Ireland). Samples from four depths were processed simultaneously, and individual filters were flash frozen in cryovials and stored at −80 °C until processing. Samples for DNA were also collected from the Scotian Shelf along the Halifax Line (HL) transect as part of the annual spring and fall Atlantic Zone Monitoring Program (AZMP; cruise codes: HUD2014004, HUD2014030, HUD2016003, HUD2016027, COR2017001, and EN2017606). Cells for AZMP DNA were collected by sequential filtration of water through 3μm and 0.2μm polycarbonate membrane filters using either a vacuum pump (2014) or a peristaltic pump (2016–2017) with coarse prefilters of 160μm (2014) and 330μm (2016–2017) (see [30] for full details).

Temperature, chl *a*, and nutrient data for the BB were provided by the Bedford Institute of Oceanography (BIO) (http://www.bio.gc.ca/science/monitoring-monitorage/bbmp-pobb/bbmp-pobb-en.php). Temperature and oceanographic data for the AZMP are also available by request from BIO. Overall, flow cytometry and molecular data covered Jan 2014–Dec 2018 and Jan 2014–Dec 2017, respectively.

### DNA extraction and sequencing

DNA was extracted using a DNeasy Plant Mini kit & protocol (Qiagen, Germany) and using the enhanced lysis procedure described by Zorz et al. [30], and then checked for amount/purity on a NanoDrop 2000c (Thermo Scientific, USA). Illumina MiSeq 300 bp paired-end sequencing of the *16S* ribosomal RNA (rRNA) gene was subsequently carried out at the Integrated Microbiome Resource at Dalhousie University as in Zorz et al. [30] and using an established microbiome amplicon sequencing workflow [31]. Duel-indexed Illumina fusion primers were used to target variable regions for bacterial *16S* V6-V8 (primers B969F & BA1406R [32]) and for universal *16S* V4-V5 (primers 515FB & 926 R [33, 34]). V6-V8 was used for AZMP samples because a partial dataset for this marker was already available for 2014 and 2016 (reported in [30] and [35]). V6-V8 sequences for 2017 have not been published elsewhere.

Amplicon sequence variants (ASVs) were determined using *QIIME 2* version *2019.7* [36] as implemented in the *Microbiome Helper* pipeline [31]. Final taxonomies were derived from a *PhytoREF*-trained classifier [37] after an initial taxonomic assignment via a full length *16S*-trained SILVA-based classifier [38] (see Supplementary Methods S2 for further details on ASV selection and characterization). BB chloroplast and cyanobacterial ASVs were rarified to 200 reads (see Fig. S2 for frequency distributions of reads per sample and Fig. S3 for rarefaction curves). Excluding samples with zero reads, at a rarefaction threshold of 200 reads there was a sample loss of ~13.5% (for V4-V5) and ~33% (for V6-V8) for surface samples (1–5 m), and furthermore, molecular datasets were skewed towards having a smaller number of final reads per sample (Fig S2). To avoid the further exclusion of samples, we did not increase the rarefaction threshold beyond 200 reads. Unless specified, rarified data were used in BB sample comparisons. AZMP ASV data were not rarefied to preserve all reads and thereby enable identification of any BB-dominant ASVs that were present but rare on the Scotian Shelf; reads for both datasets were converted to percent relative abundance scores prior to any statistical analysis or data visualization. Relative abundance = (number of reads per ASV in a sample ÷ total chloroplast and cyanobacterial reads in said sample) × 100. ASV tables and accompanying reference sequences are available as Supplementary Data S2–S4; furthermore, reference sequences for all dominant ASVs were deposited in GenBank [39] under accession codes MZ541860–MZ541862, MZ542324–MZ542326, MZ542548–MZ542554, and MZ571675–MZ571759. Raw sequencing data are also available as Sequence Read Archives listed under NCBI BioProjects PRJNA785606, PRJNA785859, and PRJNA785872 [39]. The number of sequencing reads lost during the sequence processing pipeline can be viewed in Supplementary Data S5–S7.

### Data analyses

Statistical analyses, data visualizations, and maps were generated in R version 4.0.0 [40] via RStudio version 1.2.5042 [41] using various R packages (listed in Supplementary Methods S3).

In some instances, we focused on a subset of ASVs to facilitate an in-depth assessment of species-level diversity, in such cases we report on the top twenty ASVs found in BB samples per year and representing >80% of the dataset. “Cyanobacteria” and “Phylum” (for other non-cyanobacterial ASVs) was chosen as the highest taxonomic identifiers for plotting large-scale patterns in the BB and AZMP Halifax Line (HL). For clarity, we subdivided the Ochrophyta into Bacillariophyta (i.e. diatoms), Bolidophyceae, Silicoflagellates (i.e., the Dictyochophyceae), and Pelagophyceae. The Bacillariophyta comprised diatom ASVs identified to either a specific taxonomic class (for e.g., Bacillariophyceae, Coscinodiscophyceae, etc.) or simply to the phylum Bacillariophyta. The top twenty ASVs for the AZMP were also selected but on a per sample basis given the biannual nature of this dataset. The list of top AZMP ASVs was also limited to those that reached ≥20% relative abundance in at least one sample.

*PhytoREF*-specified taxonomic assignments [37] for the top ASVs were further refined manually with online *BLAST* [42, 43] using the NCBI nucleotide (*nr*/*nt*) collection [39]. Matches closest to 100% coverage and 100% pair-wise identity (PI) were retained as the final taxonomic identification; ambiguity was resolved following priority for matches to complete genomes > complete genes > partial chloroplast *16S* rRNA (*cp16S*) gene or partial cyanobacterial *16S* rRNA gene.

Indicator species tests were run using a multi-level pattern analysis via the *multipatt* function in the *indicspecies* R package [44] using the point biserial correlation coefficient function “*r.g*” therein with 9,999 permutations.

The *MUSCLE* algorithm [45] was used to build sequence alignments to calculate in *MEGA* [46] the number of pair-wise nucleotide differences between dominant ASVs with identical taxonomies. For NMDS plots, Bray-Curtis dissimilarity scores were calculated for Hellinger standardized sample data and then NMDS was run on these data using the *vegan* package [47]. Environmental vectors were fit onto ordinations using the *envfit* function in *vegan* [47] using 999 permutations. NMDS species scores along with environmental vectors were visualized using *ggplot2* [48].

Maximum Likelihood trees that assessed the putative placement of dominant *Synechococcus* ASVs into known ecotypes for this genus [49, 50] were built using *MEGA* [46]. The distribution of dominant cyanobacterial and Euglenozoa ASVs were also compared to publicly available *Tara* Oceans datasets [51, 52] (see Supplementary Methods S4 for a more detailed explanation of how our ASVs were compared to *Tara* Oceans _mi_TAGS [52]).

Network analysis comparing V4-V5 and V6-V8 ASVs was carried out using *CoNet* [53] between identical sample sets, and the resulting network was visualized in *Cytoscape* [54] (parameter settings are given in Supplementary Methods S5).

*Vegan* [47] was used for calculating the Bray-Curtis similarities (by subtracting dissimilarity values from 1) to assess the degree of periodicity in community similarities across the 4-year time series (see refs. [55] and [56] for further information on this approach).

## Results

### Temperature and chlorophyll *a*

**Fig. 1:**
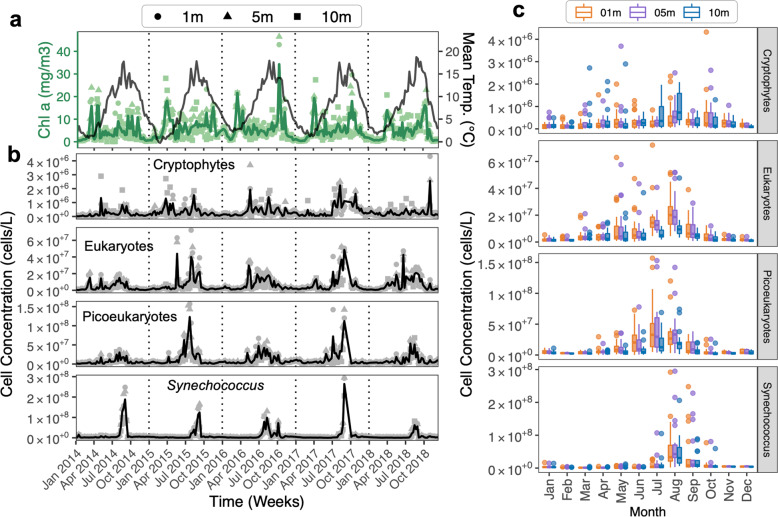
Phytoplankton counts for 5-yr (1–10 m) Bedford Basin (Halifax, NS) time series. **a** Mean chlorophyll *a* (chl *a*) and mean temperature, (**b**) mean flow cytometry cell counts for Cryptophytes, Eukaryotes, Picoeukaryotes, and *Synechococcus*. Raw data points are shown as shapes, mean is shown as black lines. **c** Monthly cell count distributions by water depth and all five years combined. The flow cytometry size range is approximately 1–35 μm. See Supplementary Methods S1 for gate descriptions. The maximum average cell density for each group was: *Synechococcus* = 2.64 × 10^8^ cells/L [mainly bloomed late August and prior to fall chl *a* peaks], Picoeukaryotes = 1.23 × 10^8^ cells/L [increased mainly during summer], Eukaryotes = 4.85 × 10^7^ cells/L [mainly increased starting late spring, then occurred throughout the summer with highest counts in August], and Cryptophytes = 2.56 × 10^6^ cells/L [bloomed mainly during summer/August and values were higher at 10 m]. Eukaryotes and Picoeukaryotes also reached higher cell counts in shallower depths (1–5 m). Highest mean chl *a* reached during the five years = 34.25 mg/m^3^ ± 18.23 SD.

The mean temperature in BB surface water from Jan 2014–Dec 2018 ranged from ~0 °C (winter months) to 18 °C (late summer) [mean minimum for surface depths (1–5 m) = −0.261 °C ± 0.15 SD: mean maximum for surface depths (1–5 m) = 18.73 °C ± 1.38 SD] (Fig. 1a). The mean surface chl *a* peaked during Mar–May in the spring and Sept–Nov in the fall (Fig. 1a). The largest chl *a* maximum (46.5 mg/m^3^) was in fall 2016 (Fig. 1a). Annual increases in chl *a* during fall and spring blooms were similar between the two seasons, except for 2016, when the fall increase was markedly larger [Fall 2016 = 46.5 mg/m^3^ at 5 m versus Spring 2016 = 21 mg/m^3^ at 1 m] (Fig. 1a).

### Broader taxonomic groups and flow cytometry

**Fig. 2:**
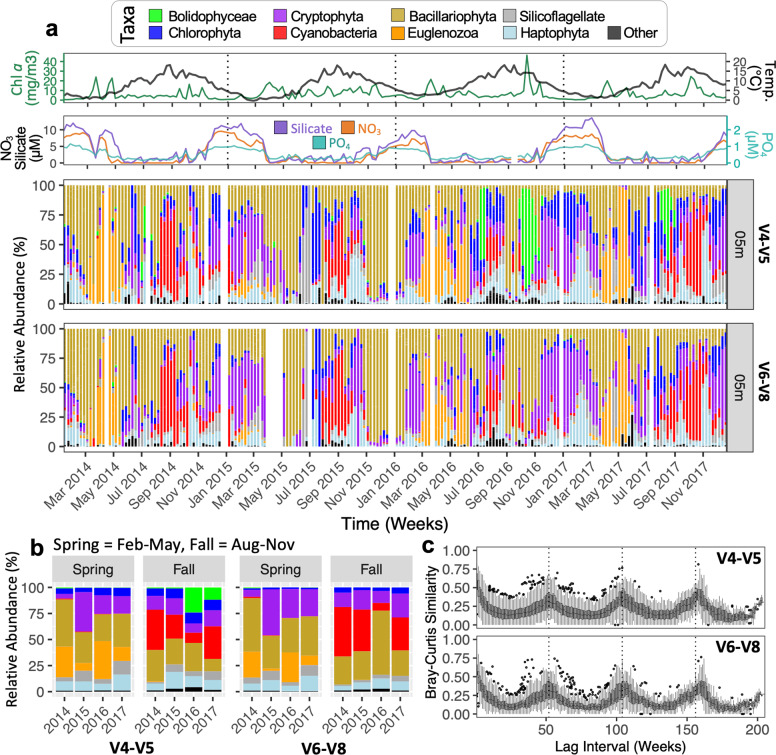
Weekly dynamics and seasonal patterns for major phytoplankton groups observed over the 4-yr time series in Bedford Basin (Halifax, NS). Sequences are chloroplast *16S* plus cyanobacterial *16S*. **a** weekly relative abundances for all ASVs at 5 m based on their assignment to a major taxonomic group; values shown relative to nutrients, chl *a* and temperature [see Fig. S5 for 1 & 10 m depths]. Unrarefied data (shown in figure) had identical trends to rarefied data, yet unrarefied data retained more samples [compare Figs. S5, S6]. White columns represent missing or unsuccessfully sequenced samples, or those that only had bacterial *16S* reads; for our 1–60 m Bedford Basin datasets 9 (V4-V5) and 23 (V6-V8) samples only yielded bacterial *16S* data, and 2 (V4-V5) and 5 (V6-V8) samples did not yield any final sequence reads post processing (Supplementary Data S5–S7). **b** Summary bar-plots comparing total relative abundance of broader groupings per season and year [going from Feb-01 to May-01 and Aug-01 to Nov-01], data shown are rarified to 200 reads and include all ASVs at 1–10 m depths. **c** Distributions of Bray-Curtis similarities between 5 m samples plotted against the number of weeks separating samples [relative abundances used from rarefied data; values were also Hellinger transformed [47]].

ASVs belonged to eight broader taxonomic groups (Fig. 2a), which generally overlapped between the two genetic markers used. The most notable exception was the Bolidophyceae (mainly observed for V4-V5 and at lower percentages for V6-V8; Fig. 2a). We also assessed the similarity for taxonomic assignments between V4-V5 and V6-V8 using a network analysis; for ~64% of top ASVs that co-occurred with another top ASV in a one-to-one relationship (between opposite markers) the two variable regions led to the same species name (Fig. S4 and Supplementary Results S1). Note the ‘top 20’ (referred to as ‘top’) BB ASVs are those exhibiting the highest annual relative abundances [82% and 80% of all BB V4-V5 and V6-V8, respectively (for chloroplast and cyanobacterial *16S* reads)]. Molecular interannual comparisons at 5 m for all ASVs (Fig. 2) indicates that: (i) Bolidophyceae displayed higher relative abundances in 2016 & 2017; (ii) Haptophyta displayed higher relative abundances primarily in winter/preceding the spring chl *a* maxima and sometimes near the fall chl *a* maximum (e.g. 2015)—the winter period also showed low chl *a*, as well as low Eukaryote and Picoeukaryote cells via flow cytometry (gates that would include haptophytes; Figs. 1 and 2a), suggesting that colonial haptophytes too large to be captured by flow-cytometry (e.g. *Phaeocystis* [57]) may have been present; (iii) Chlorophyta, Bacillariophyta, and Silicoflagellates displayed less consistent trends in relative abundances corresponding to seasonal phytoplankton blooms; (iv) Cryptophytes were consistently present during months with higher nutrient concentrations; and (v) cyanobacteria and Euglenozoa usually dominated the phytoplankton community during/near the fall and spring bloom periods, respectively. For top BB ASVs *Synechococcus* was the only genus within the cyanobacteria. Unrarefied relative abundances between V4-V5 and V6-V8 were strikingly similar apart from the Bolidophyceae (Fig. 2a). Corresponding data for 1 m and 10 m BB depths also showed similar results (Fig. S5) and rarified data versus nonrarefied data showed near identical trends (Figs. S5, S6).

Several groups of phytoplankton (e.g., Bacillariophyta) did not display peak relative abundances consistently corresponding to seasonal blooms, but rather displayed higher relative abundances intermittently throughout the time series (Fig. 2a). The relative abundances for each major taxonomic group for all ASVs from 1–10 m depths with respect to spring and fall months are shown in Fig. 2b. While the dominant taxonomic group in the spring months tended to differ annually, cyanobacteria were typically the dominant group in the fall (Fig. 2b), suggesting that the phytoplankton groups dominating in the spring in BB may be less predictable than in the fall. Flow-cytometry counts further showed that the BB phytoplankton community displayed an increase in several microalgal groups after the spring bloom and into the summer months, with *Synechococcus* increasing prior to late fall and winter months (Fig. 1b). Specific flow cytometry estimates of cell densities for Cryptophytes, Eukaryotes, Picoeukaryotes, are also shown (Fig. 1b, c). While we did not observe recurrent increases in cell density for any particular group prior to the increases in chl *a* corresponding to spring blooms (Fig. 1), we did observed peaks in Eukaryotes (2014) and several peaks in Cryptophytes (2015) that correspond to chl *a* increases in those respective years (Fig. 1). Flow-cytometry results were remarkably consistent with historical records (see http://www.bio-iob.gc.ca/science/monitoring-monitorage/bbmp-pobb/bbmp-pobb-en.php; last accessed 7-May-2020) with maximum values for *Synechococcus*, Picoeukaryotes, and Cryptophytes, as well as temperature and chl *a* maxima ranges being comparable to previous studies [5, 9, 24, 58]. As previously observed [9], the spring bloom may have been populated by larger phytoplankton cells that were not recorded by flow cytometry. As our DNA sequencing indicates, the phytoplankton community dynamics during the spring period appear to be better resolved using molecular approaches (Fig. 2).

Although weekly trends suggested that finer scale changes in phytoplankton community compositions even for very broad taxonomic groups could occur quite rapidly from week-to-week (Fig. 2a), an analysis of the Bray-Curtis similarities between samples relative to the number of weeks between samples (i.e. the lag interval) verified an underlying year-to-year community stability/cyclicity for the phytoplankton of Bedford Basin (observe similarity increases at ~52-week intervals; Fig. 2c) [55]. This trend was also consistent for all three surface depths, for both *16S* markers, and regardless of whether sequence data was rarefied or not (Figs. S7, S8).

The Euglenozoa reoccurred near/during the spring bloom and showed an increase in relative abundance that generally paralleled the increase in chl *a* in 2014, 2016, and 2017 (they also remained present for several weeks after spring chl *a* peaks; Fig. 2a). Interestingly, the lower relative abundances of Euglenozoa in 2015 was balanced by a larger relative abundance and cell counts for Cryptophyta (Figs. 2a, 1b, respectively). Given that *Synechococcus* and Euglenozoa were the only broader taxonomic groups that displayed clear temporal profiles linked to fall and spring bloom periods, respectively, we designated these two groups as indicator species of seasonal BB phytoplankton blooms (Table S1). Given their regional importance, we searched for the top ASVs belonging to these two groups within _mi_TAGs from *Tara* Oceans data [51, 52, 59]. BB *Synechococcus* (V4-V5: *n* = 2) and Euglenozoa (V4-V5: *n* = 1) have matches to the *Tara 16S*_mi_TAGs (Fig. S9) [51, 52]; these trends suggest that the two top BB *Synechococcus* ASVs are found globally (i.e. they are likely cosmopolitan), while the top Euglenozoa ASV was only detected at two *Tara* sites, the North Atlantic (39.2305, −70.0377) and the Southeast Atlantic shelf waters (−32.2401, 17.7103) suggesting a potential preference for coastal (or near coastal) regions [51, 52] (Fig. S9).

### Individual ASV Profiles

**Fig. 3:**
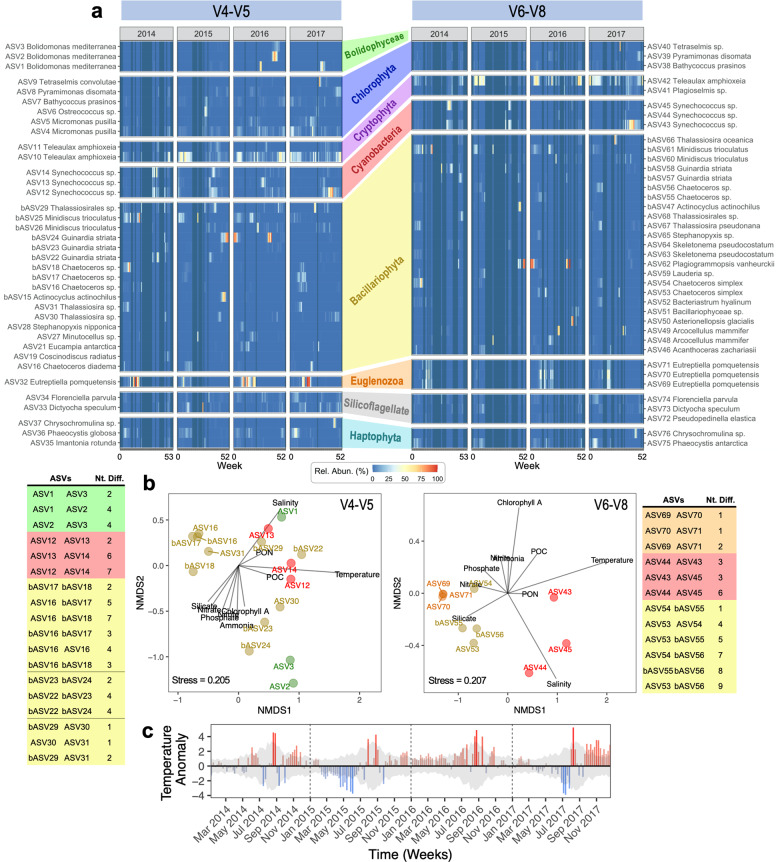
Individual temporal relative abundances profiles for Top Twenty phytoplankton *16S* ASVs (chloroplast + cyanobacterial *16S*) in the Bedford Basin time series from 2014–2017. Data is rarefied. **a** Weekly relative abundances (Rel. Abun.) at 5 m water depth for V4-V5 and V6-V8. Rel. Abun. (%) = (number of reads per ASV in a sample / total chloroplast and cyanobacterial reads in said sample) × 100. **b** Plot of species scores after 5 m samples subjected to NMDS analysis. Only groups of ASVs with >3 identical genus and/or species names are shown along with the number of nucleotide differences (Nt. Diff.) between these ASVs. Environmental variables are overlaid as lines. **c** Weekly temperature anomalies at 5 m [values are ± °C difference relative to the 1992–2017 weekly mean temperature]. Shading in anomaly plots show standard deviations of the weekly 1992–2017 means. Anomalies for nutrients and salinity also given (Fig. S11). Also see the indicator species test of Table S1 for significant seasonal associations of top ASVs.

Weekly relative abundance profiles for the top twenty BB ASVs are shown (Fig. 3a); for V4-V5 *n* = 37 ASVs and V6-V8 *n* = 39 ASVs [*n* can be >20 due to yearly differences]. Comparison to *PhytoREF* [37] provided broader taxonomy (i.e., typically class-level); however, 59% of V4-V5 and 74% of V6-V8 ASVs required further comparison to the NCBI *nr*/*nt* database to obtain a species or genus assignment [39]. For consistency, the classification for each top ASV was confirmed via BLAST in NCBI [43].

Some ASVs occurred in select years and sporadically throughout the year (e.g. ASV4 *Micromonas pusilla* and ASV7 *Bathycoccus prasinos*; Fig. 3a), while others occurred at high relative abundances and displayed a consistent annual pattern (e.g. ASV11 *Teleaulax amphioxeia*, *Synechococcus* sp. [ASVs 12, 14, 43, & 45], *Chaetoceros* sp. + *Chaetoceros simplex* [bASVs 17, 18, 55, 56 & ASV54], *Eutreptiella pomquetensis* [ASV32/ASV69–71], and *Phaeocystis* spp. [ASV36/75]) (Fig. 3a). Seasonally early (Spring + Summer) and late (Summer + Fall) *Minidiscus trioculatus* ASVs were also observed (Fig. 3a; Table S1). We consider that indicator taxa (Table S1) that returned frequently and regularly during the 4-yrs likely provide the optimal metric for overall phytoplankton community change in our temperate coastal waters.

Multiple top ASVs were often identified within the same species or genus. NMDS analyses were used to assess the preferred environmental conditions for closely related ASVs to determine whether sequences with only 1–9 bp differences (Nt. Diff.; Fig. 3b) represented true biological variants or perhaps resulted from sequencing errors [60]. The distribution similarity of closely related ASVs over the 4-yr period at 5 m was plotted using NMDS ordination overlaid with environmental variables to determine whether the ASVs co-varied temporally (Fig. 3b). This analysis revealed that small V4-V5 and V6-V8 chloroplast and cyanobacterial *16S* nucleotide differences could represent true interspecific differences with ecological relevance (see Supplementary Results S2 for more specific trends).

Changes in temperature appeared to influence the temporal patterns of several ASVs designated as key indicator species (Fig. 3). In particular, *Synechococcus* ASV12 & ASV43 had higher relative abundance values during 2017 when there were consistently high temperature anomalies during the late fall/early winter (Fig. 3c). The opposite was seen for *E. pomquetensis*, whereby its temporal patterns were consistent with the laboratory-determined narrow growth range of 0–10 °C for this species [61](Fig S10). *E. pomquetensis* (ASV32 & 69–71) had especially low relative abundances during 2015, which was the only year with 5 m temperature down to 0 °C (Figs. 1a, 2a, and S10). We propose that the sub-zero temperatures reached at 5 m during spring 2015 led to the observed shift from Euglenozoa to Cryptophytes (Fig. 2a). The patterns above also lend their support to the use of *Synechococcus* ASV43 and *E. pomquetensis* as indicator species in BB, given that changes in the relative abundance of these two phytoplankton groups paralleled temperature anomalies (warmer and cooler conditions, respectively; Fig. 3).

For the ASVs identified to at least genus-level we provide summary stats and reference accessions for *BLAST* matches (Table S2) [43]. Nearly all the dominant phytoplankton identified to species-level were marine (according to www.algaebase.org, last accessed 17-May-2021), except for *Acanthoceras zachariasii* (freshwater) [62] and *Pseudopedinella elastica* (brackish) [63]. Hence, freshwater input appeared to have little influence on shaping the dominant phytoplankton observed.

### Comparisons to the Scotian shelf

**Fig. 4:**
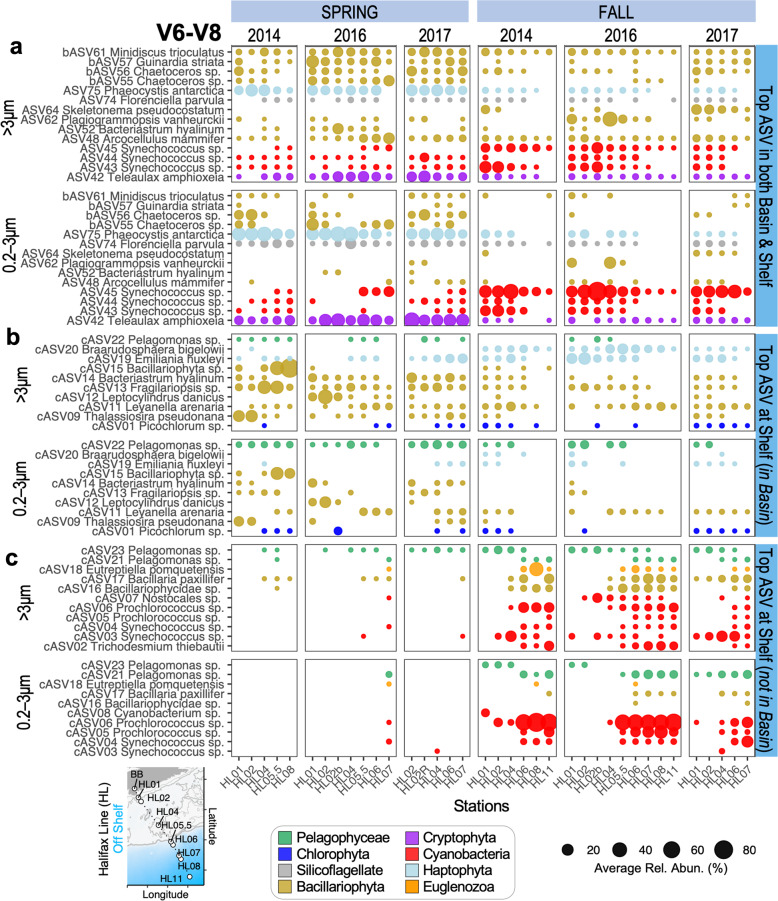
Comparison of top Scotian Shelf phytoplankton ASVs versus ASVs that were observed in the Bedford Basin (BB) time series. Stations were sampled from the Halifax Line (HL) transect during Spring and Fall Atlantic Zone Monitoring Program (AZMP) expeditions. **a** ASVs that are within the top twenty list for both Basin and Shelf. **b** Top AZMP ASVs that were also detected in the Bedford Basin; however, they are not among the top basin-specific ASVs. **c** Top AZMP ASVs that were not detected in the Bedford Basin. Note that AZMP ASVs are limited to the top twenty most relatively abundant ASVs per sample, and to those ASVs that reached ≥20% in at least one sample. No AZMP cruise data available for 2015. Individual relative abundances or ‘Rel. Abun.’ as % = (number of reads per ASV in a sample / total chloroplast and cyanobacterial reads in said sample) × 100; an average was then taken per station. Data are from V6-V8 metabarcoding between 1 and 80 m water depths (surface & photic zone [30]); data unrarefied. Summary stats for BLAST results given in Table S3. Stations HL02b and HL02R were second samplings of HL02 (during return trips to shore). Note stations are organized left-to-right by increasing distance from the Bedford Basin.

Using spring and fall AZMP data, we found that the vast majority [85% or 33/39] of the top V6-V8 BB ASVs were present on the Scotian Shelf (Fig. S12). The six top BB ASVs in the fjord that were not found on the Scotian Shelf during our study were ASV40 *Tetraselmis* sp., ASV69-ASV71 *Eutreptiella pomquetensis*, ASV73 *Dictyocha speculum*, and bASV60 *Minidiscus trioculatus*. At the shelf there were a total of 36 top AZMP ASVs: 66% of these were also recovered in the fjord, 39% were dominant in both regions, 28% were dominant on the shelf but still found in the fjord, and 33% were dominant at the shelf but absent in the fjord (Fig. 4).

Two BB ASVs [ASV42 + ASV75] had especially high relative abundances across samples from nearly all shelf stations in the spring (Figs. 4 and S12), confirming the importance of *T. amphioxeia* and *Phaeocystis* sp. to phytoplankton communities inside the fjord, as well as beyond the shelf break during spring periods (Figs. 3, 4, and Table S1). Similarly, ASV45 *Synechococcus* sp. was observed in the small size fraction at consistently high relative abundances across nearly all AZMP HL stations in the fall season (Fig. 4). Maximum-Likelihood trees indicate that the BB *Synechococcus* likely belong to *Synechococcus* clades I (ASV13 and ASV44) and IV (ASV14; Fig. S13) [49, 50].

Analysis of top AZMP ASVs associated with the shelf (Fig. 4) revealed that: (i) cASV20 *Braarudosphaera bigelowii* and cASV13 *Fragilariopsis* sp. were consistently dominant in the >3μm fraction at all HL stations in the fall and spring, respectively (these variants were also identified in the fjord but outside its list of top ASVs), (ii) *Pelagomonas* sequence variants (cASV22 & 21) are important throughout the shelf, and (iii) dominant AZMP ASVs, which are also absent from the fjord, were mainly found during the fall season beyond the shelf break (for e.g., *Trichodesmium* and *Prochlorococcus*). This last class of ASVs also included an off-shelf fall-associated *E. pomquetensis* variant (cASV18) that was not observed in the fjord (Fig. 4c); this ASV likely represents a warm-water associated ecotype given that it occurred in 20.2 ± 1.7 °C waters at station HL08.

From a broader perspective, AZMP data revealed that numerous phytoplankton species observed by the BBMP are also found on the Scotian Shelf and often during both spring and fall seasons (Figs. 4 and S12). Hence, these taxa are key phytoplankton beyond the Bedford Basin and into the more expansive coastal NWA shelf waters.

### Small phytoplankton and their link to atypical temperature conditions

**Fig. 5:**
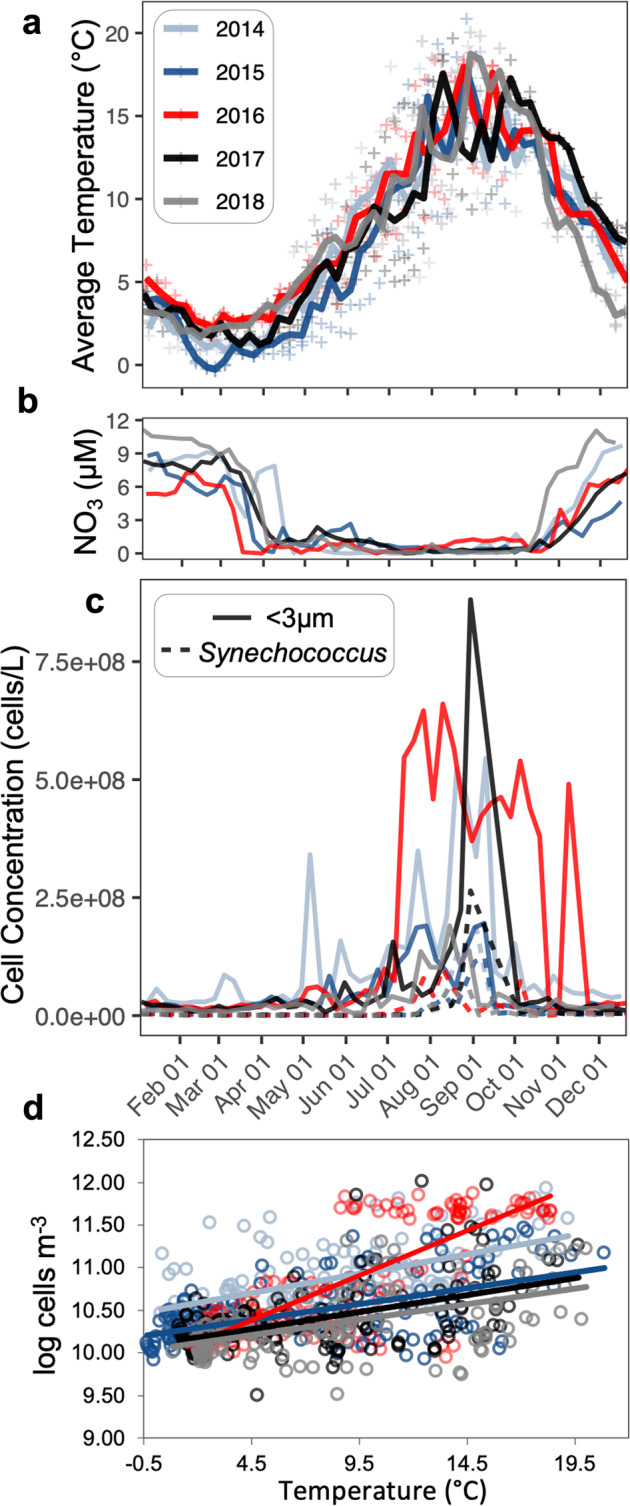
Warmer temperatures and trends for smaller phytoplankton during 2016. **a** Average surface temperatures by year [raw data shown as plus symbols], (**b**) surface nitrate levels by year suggestive of weaker winter mixing in 2016, (**c**) average surface <3μm cell concentrations (plotted by Month/Day), and (**d**) their yearly correlations with temperature (for the <3um group). Surface depths were 1 m, 5 m, and 10 m samples. Trendlines in (**d**) assumed a linear relationship, and the equations: 2014: y = 0.0454x + 10.495; 2015: y = 0.0369x + 10.222; 2016: y = 0.1051x + 9.9062; 2017: y = 0.0401x + 10.099; and 2018: y = 0.0366x + 10.04. For comparison, the 1993–2005 cell density vs. temperature relationship for BB phytoplankton (not just <3μm) is reported as y = 0.097x + 9.47 by [5]. Individual years are shown in Supplementary Fig. S15.

Based on the example of a temperature related community shift from *Eutreptiella* to cryptophytes (Figs. 1, 2) and historical observations linking temperature and cell density [5], we examined the relationship between temperature and <3μm cells. Temperature versus cell densities from 2014–2018 demonstrated that in 2016 abnormally high temperatures throughout winter, summer, and early fall months (Figs. 3c and 5a) coincided with higher densities for <3μm cells throughout the summer and fall (Figs. 5c and S14). Nitrate levels were low during the winter mixing of 2015/2016, amounting to a period of consistently low nitrate anomalies (Figs. 5b and S11). This same year also displayed an increase in the correlation between temperature and density for <3μm cells (Fig. 5d). Note that another study has already proposed weaker mixing in the Bedford Basin during Winter 2015/2016 (see ref. [64]). In addition to flow cytometry counts, *cp16S* and cyanobacterial *16S* data for 2016 further indicated the presence of smaller phytoplankton (Table 1). Lastly, an examination of shelf data also hinted that the unique dynamics of 2016 may not have been restricted to the fjord alone; for instance, ASV62 *Plagiogrammopsis vanheurckii* occurred at every station along the HL transect in Fall 2016 (Figs. 4 and S12).

**Table 1:** Small taxa associated with trends in 2016 (values are for 1–10 m; unrarefied data).

ASV	Ave. Rel. Abun. (% ± SD) [Period]	Approximate Cell Sizes (μm^3^)
ASV49 *Arcocellulus mammifer*	62 ± 20 [Early Jul]	~11^a,*^
ASV44 *Synechoccocus* sp.	21 ± 18 [Late Jul]	~0.38–1.15^b^
	16 ± 11 [Early Aug]	
ASV42 *T. amphioxeia*	21 ± 10 [Late Jul]	~109^c^
	10 ± 8 [Early Aug]	
ASV62 *Plagiogrammopsis vanheurckii*	61 ± 8 [Last Week of Aug]	~372^d^
	56 ± 35 [Sept]	
ASV50 *Asterionellopsis glacialis*	68 ± 18 [Oct]	~1,492^c^

^a^Using dimensions in [87] and formula in [88].

^b^See [89, 90].

^c^Median of volumes reported at nordicmicroalgae.org (last accessed 14-Apr-2021).

^d^See [91, 92].

^*^Network (Fig. S4) suggested ASV49 *A. mammifer* may be ASV1 *B. mediterranea*, however, even if the latter is correct, cells would still be small given *B. mediterranea*’s diameter of 1–1.7 μm [93]. Furthermore, *A. mammifer* is reported as ≲3μm in its shorter axes [87].

## Discussion

### *Synechococcus* and *Eutreptiella* are important phytoplankton *16S* rRNA gene signatures in the Bedford Basin

Photosynthetic organelles along with their plastid genomes [65] have garnered attention as targets for characterizing phytoplankton communities [37, 66, 67]. Overall, our chloroplast plus cyanobacterial *16S* metabarcoding approach revealed a coherence in the multiyear phytoplankton community composition, i.e., some similarity to previous microscopy records, while also providing higher resolution for species- (and in some cases) ecotype-level taxonomy for smaller phytoplankton (Supplementary Discussions S1, S2 provide further context and information on this topic).

*Synechococcus* cyanobacterial patterns were the most consistent feature present between flow cytometry and chloroplast/cyanobacterial *16S* data during our study. Although some *Synechococcus* ecological patterns for the Bedford Basin were previously known [5], we expanded this knowledge by demonstrating that the dominant BB *Synechococcus* ecotypes belong to clades I and IV. Others have recently demonstrated a shift from *Synechococcus* near the fjord to *Prochlorococcus* off-shelf [30].

In contrast to *Synechococcus*, our molecular data demonstrated Euglenozoa, particularly *E. pomquetensis*, had a distinct spring/early summer occurrence pattern at our study site. Although the basic biology of *E. pomquetensis* is known [61], to the best of our knowledge, the striking patterns of this species’ association with the spring period has not previously been described. This is possibly due to sampling design and/or issues with morphological identification, which may in some cases obfuscate this trend (Supplementary Discussion S3). Given that Euglenozoa was observed at high relative abundance even after chl *a* maxima (e.g. 2016; Fig. 2) and that *16S* data is compositional, one can suggest that Euglenozoa may retain a presence after the bloom via a mixotrophic lifestyle that would include grazing in addition to photosynthesis, thereby explaining why Euglenozoa remained present weeks after peaks in spring chl *a* when inorganic nutrient availability decreases. Confirmed mixotrophy in another *Eutreptiella* species [68] suggests that *E. pomquetensis* could have a mixotrophic lifestyle.

Overall, we propose ASV43 *Synechococcus* (Clade I) and ASV32/69-71 *E. pomquetensis* as indicator species having special importance for detecting environmentally driven change in the fjord for fall and spring seasons, respectively, as the former was linked to warmer summer temperatures, while the latter appeared adversely affected by colder winter/spring temperatures [61] (Fig. 3). Changes in these particular ASVs might possibly be a preamble to trends expected from ongoing climate change. Ultimately the molecular identification of seasonally specific indicator species provides a framework and baseline from which to assess (through DNA sampling) any future effects that extreme environmental change may have on the typically reoccurring primary producers of the coastal NW Atlantic.

### Phytoplankton tracked by the Bedford Basin time series are globally relevant

Although there has been interest in the bacterial communities along the Scotian Shelf [30, 35], the phytoplankton communities remain relatively unexplored via molecular analysis except for recent reports by Zorz et al. [30] and Willis et al. [35], as well as previous *18S* sequencing by Dasilva et al. [69].

Recent literature suggests that many of the phytoplankton we identified within the Bedford Basin, and nearby at the Scotian Shelf, are globally significant. *Synechococcus* (of clades I & IV in the fjord), were previously identified as important in the NWA and were especially dominant in the subpolar region during winter [6]; members of these clades have also been detected in colder waters with elevated nutrients in the North Pacific Ocean [49]. Several phytoplankton inhabiting the fjord were similarly reported on the Scotian Shelf by others [69] and in the off-shore NWA [6]. Those in common include: *Bathycoccus*, *Micromonas*, *Chaetoceros*, *Phaeocystis*, *Teleaulax*, and *Thalassiosira*, which were all identified as key phytoplankton found in the off-shore NWA with both *Micromonas* and *Bathycoccus* especially relevant to the NWA subtropic zone [6, 70]. Members of these two taxa are also widespread [71], and *Micromonas* and *P. antarctica* both occur in the Southern Ocean [72]. In addition to genera mentioned above, *Ostreococcus*, *Dictyocha*, *Florenciella, Fragilariopsis, Minidiscus*, and *Braarudosphaera bigelowii* were also observed on the Scotian Shelf [69]. *Florenciella parvula* has been reported as an important component of the dictyochophyte fraction in mesotrophic surface samples from the eastern North Pacific [73], while the shelf-wide distributed *Minidiscus trioculatus*, was recently proposed as a small diatom of great importance due to its widespread occurrence and likely contribution to carbon export [74]. *Fragilariopsis* and *B. bigelowii* are also of special note; the former is common in polar environments [75], while the latter is associated with the symbiotic nitrogen-fixing unicellular cyanobacteria, *Candidatus* Atelocyanobacterium thalassa [76, 77]. Furthermore, the *Pelagomonas* sp. identified herein matched known (and geographically wide-ranging) wildtype *Pelagomonas* [78]. Interestingly, *T. amphioxeia* was recently reported to have two morphotypes with differing ploidy and winter/spring versus summer distributions [79]. BBMP data may reflect this novel ecology; however, additional data/confirmation is needed. Finally, *Arcocellulus mammifer*, one of the small Bacillariophyta we recognised as important in 2016, responded positively to increased temperature in field incubation experiments conducted at the San Pedro Ocean Time-series (from NE Pacific) [80]. Like *A. mammifer*, several small taxa (Table 1) were linked to the unique dynamics of 2016; future experimental work should investigate these taxa from the perspective of community-based growth responses under warming in situ temperatures.

Cell counts and community composition-based analyses for <3μm cells versus temperature demonstrated that in the coastal NWA small phytoplankton are particularly responsive to increases in in situ temperatures during the same year. This trend was likely not a sole consequence of temperature, but rather temperature’s effect on the water column during earlier parts of 2016, and therefore nutrient availability. This is supported by Haas et al. [64] who concluded weaker BB winter mixing during 2015/16, as well as by observations herein of negative nitrate anomalies (Fig. S11). While the generally accepted view of higher small phytoplankton cell densities in late summer and fall for the BB/NWA was upheld during the span of our study [5], the unique pattern of 2016 (effectively a higher temperature and lower nutrient scenario) highlights the variable nature of the cell density versus temperature relationship within the coastal NWA (particularly under warmer conditions) [5]. This variability ultimately points to high-frequency sampling and datasets as being essential for identifying changing trends within coastal environments of the NWA.

Collectively, our results established a baseline for seasonal variation in phytoplankton *16S* rRNA gene diversity over a period of several years in the Bedford Basin, a coastal fjord that has been sampled for several decades [9]. Many of the phytoplankton tracked by the BBMP time series are globally relevant, hence our observations provide highly resolved data for some of the most important oceanic primary producers. In essence, the phytoplankton community of the BBMP is a continuum of the phytoplankton in the NWA and shows important weekly trends for species that are dominant in the NWA, including, *Arcocellulus*, *Bolidomonas*, *Teleaulax*, *Minidiscus*, *Chaetoceros*, *Phaeocystis*, as well as multiple ecotypes of *Synechococcus*. As such, the results presented herein contribute to our known understanding of the biota within the NWA—a region of global significance for marine productivity, sustainable marine fisheries [81, 82] and predicted global phytoplankton richness [19].

### Additional insights gained from high-frequency DNA sampling

Ocean time series continue to be a key resource for the study of ocean microbiomes and their community dynamics [55, 83]; for instance, our ability to track phytoplankton with weekly frequency provided additional insights into: (i) the existence of potentially novel/unknown ecotypes (for e.g., a warm-water associated *Eutreptiella* ASV at the edge of the Scotian Shelf), (ii) the extent to which weekly community transitions can occur for dominant phytoplankton within the region (for e.g., rapid transitions in phyla-level community compositions were often evident even within monthly timeframes), and (iii) the general utility of both V4-V5 and V6-V8 within the cp*16S* rRNA gene for tracking phytoplankton (as corroborated by our network analysis between the two markers). As these points suggest, the ecological knowledge that can be gained from high-resolution molecular sampling of the ocean microbiome using a stationary time series is multifaceted and can range from the characterization of basic species distributions to the collection of in situ observational data that can reveal ocean variability on multiple time scales [83].

Another major advantage of weekly DNA sampling is that our final time series provided insight into the phytoplankton successional trends (that is the restructuring of community compositions across time; [84]) that occurred over four complete annual cycles. Classically, phytoplankton succession in the NWA has been defined by reoccurring yearly cycles of pico-phytoplankton (prior to spring bloom), diatoms (during the spring bloom), followed by other phytoplankton (e.g., coccolithophores post spring-bloom), with further succession towards small phytoplankton during the fall bloom [5, 6, 85]. When we examined Bray-Curtis similarities between samples, we observed a clear cyclical relationship for phytoplankton communities, indicating that there is indeed an underlying reoccurring cycle with respect to the in situ phytoplankton diversity of the Bedford Basin (Fig. 2c; ref. [55]). Another feature of this pattern, however, was that peaks in Bray-Curtis similarities typically only approached 0.5 (as opposed to 1.0 for 100% identical communities; [55]); therefore, despite the phytoplankton community displaying cyclicity over multi-year scales, the patterns were not entirely deterministic (i.e., non-random; [86]) in the sense that the community composition was not exactly the same each year [55]. This point, along with the various individual temporal profiles we presented for dominant phytoplankton ASVs within the Bedford Basin (Fig. 3) demonstrates the rather complex nature of the in situ phytoplankton diversity that exists within the classical succession patterns mentioned earlier. For example, obvious and repeatable patterns in *16S* rRNA gene relative abundances were observed for *Phaeocystis* (appearing in early spring), *Eutreptiella* (appearing in spring and early summer), and *Synechococcus* (appearing in fall). While in contrast, the successional patterns for diatoms were more semi-predictable in that dominant diatom ASVs appeared year-after-year, but their temporal reoccurrence patterns were less clearly defined. Given that detailed in situ molecular observations can be lacking for key transitional periods such as during spring blooms [85] and during winter periods [6], in future, it will be worthwhile to use our molecular time series of phytoplankton diversity to inform additional studies regarding phytoplankton succession within the coastal NWA.

## Conclusions

In this study we presented a detailed time series of phytoplankton occurring at a coastal site within the Northwest Atlantic, along with coincident phytoplankton observations at a nearby transect along the Scotian Shelf. Together these datasets: (i) provided a comprehensive and broad survey of the dominant phytoplankton within the coastal NWA across all four seasons, (ii) revealed the identity of key indicator species and novel ecotypes within the region, (iii) pointed towards the contribution of smaller cells under anomalous nutrient and temperature conditions, and (iv) validated the use of two *16S* rRNA gene variable regions (V4-V5 & V6-V8) for phytoplankton tracking and for investigating intraspecific (e.g. ecotype) patterns in the context of time-series molecular data. Collectively, our analyses amount to a more detailed molecular picture of both the cumulative and seasonal phytoplankton biodiversity within the coastal NWA. Our ability to link regionally-specific taxa to the phytoplankton present at the Scotian Shelf and within the global ocean via literature further supports the view that the Bedford Basin Monitoring Program (BBMP) is especially well suited for identifying seasonal and interannual trends for a variety of key temperate phytoplankton. Therefore, with its ease of access and long-term high-resolution set of observations, the BBMP is an initiative that lends itself as a perfect backdrop for further manipulative field experiments and process studies to assess the future effects of climate change on primary productivity in the NWA.

## Supplementary information

Supplementary Figures

Supplemental Text and Supplemental Tables

Supplemental Data S1

Supplemental Data S2

Supplemental Data S3

Supplemental Data S4

Supplemental Data S5

Supplemental Data S6

Supplemental Data S7
