# Supplementary material for: Highly-resolved interannual phytoplankton community dynamics of the coastal Northwest Atlantic
Source: ISME Commun. 2022 Apr 20;2:38. doi: 10.1038/s43705-022-00119-2 (PMC9723599; doi:10.1038/s43705-022-00119-2)
Supplement: Supplementary file 1 — Supplementary Figures [file 43705_2022_119_MOESM1_ESM.pdf]

SUPPLEMENTAL FIGURES for

Highly resolved interannual phytoplankton community dynamics of the coastal Northwest Atlantic

Brent M. Robicheau[1,*], Jennifer Tolman[1], Erin M. Bertrand[1], Julie LaRoche[1,*]

[1]Department of Biology, Dalhousie University, Halifax, NS, Canada

[*]Corresponding author emails: brobicheau@dal.ca, Julie.Laroche@dal.ca

Included in this PDF file:

Figures S1–S15

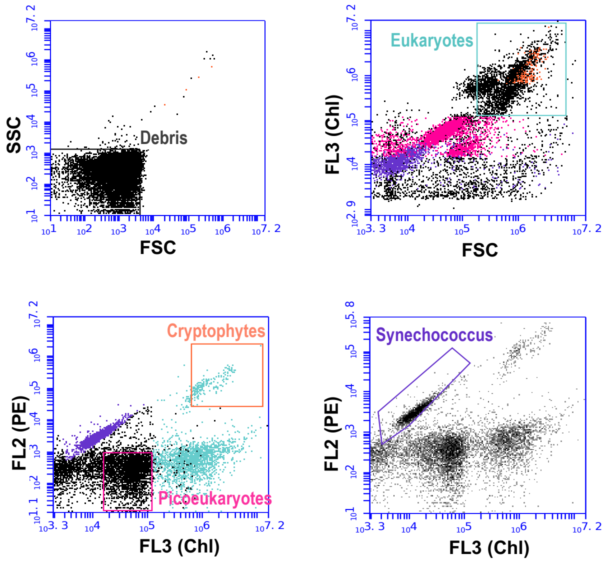

Fig S1. Diagram of flow cytometry gates used. See supplemental methods for further details about gate descriptions. Debris gate shown using a negative control.

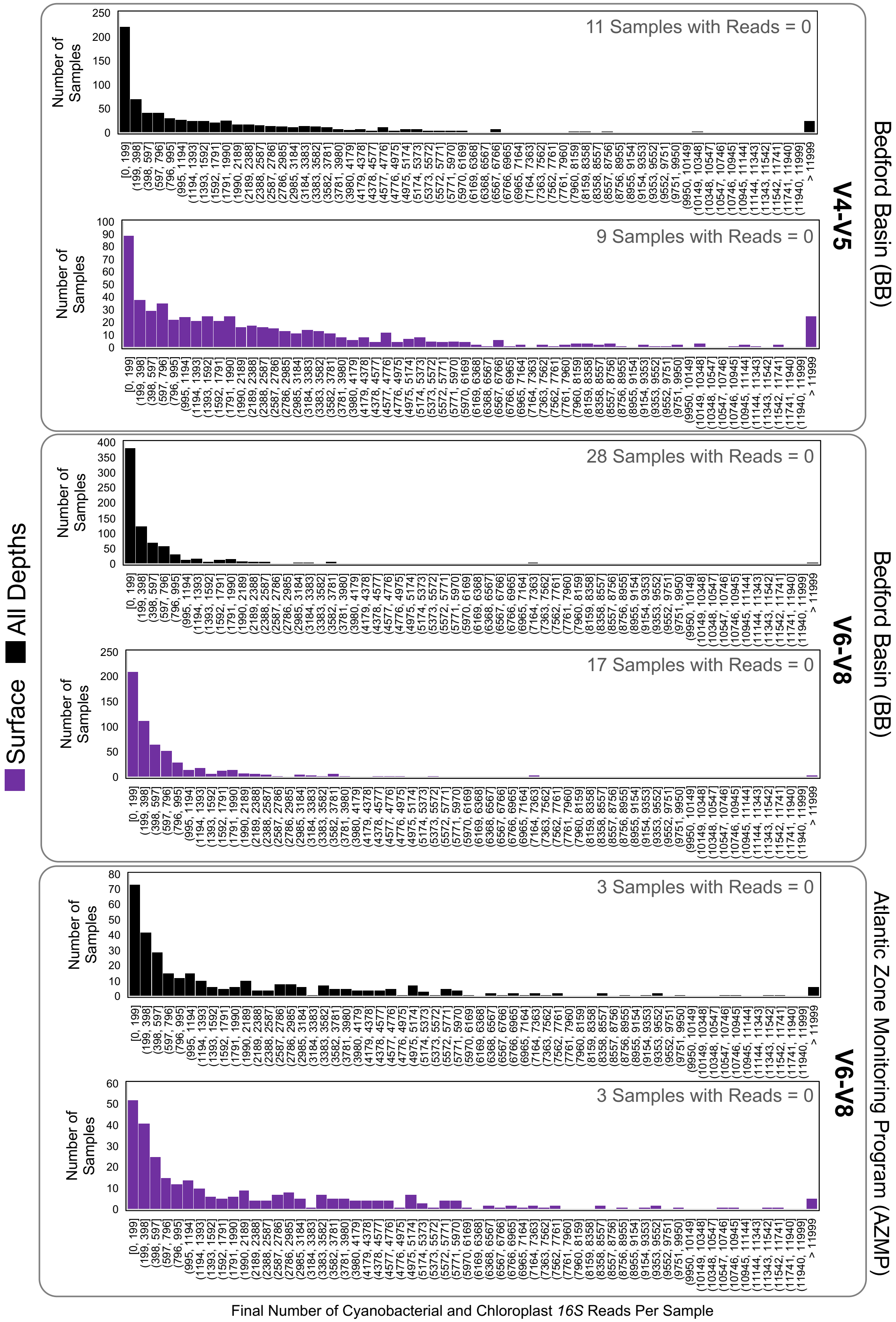

Fig S2. Frequency distributions for the final number of chloroplast & cyanobacterial *16S* reads per sample. BB surface = 1-10m; AZMP surface = 1-80m (includes photic zone).

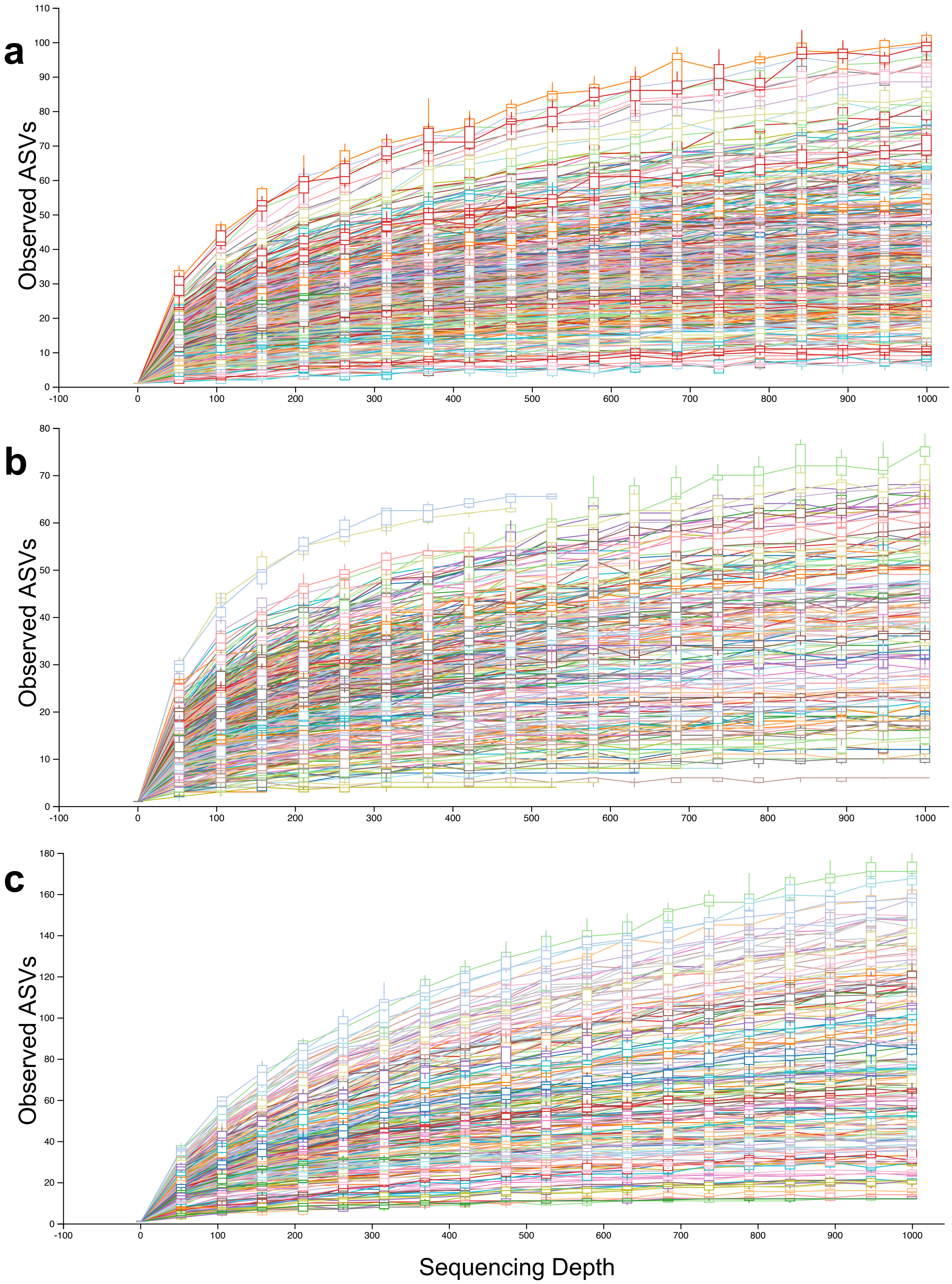

Fig S3. Rarefaction curves for the three *16S* rRNA datasets used in our study. Panels corresponding to datasets for: (**a**) Bedford Basin (BB) V4-V5, (**b**) BB V6-V8, and (c) Atlantic Zone Monitoring Program V6-V8. Colors represent samples; no legend shown as colors can repeat for different samples. Plots generated using QIIME 2 View [36].

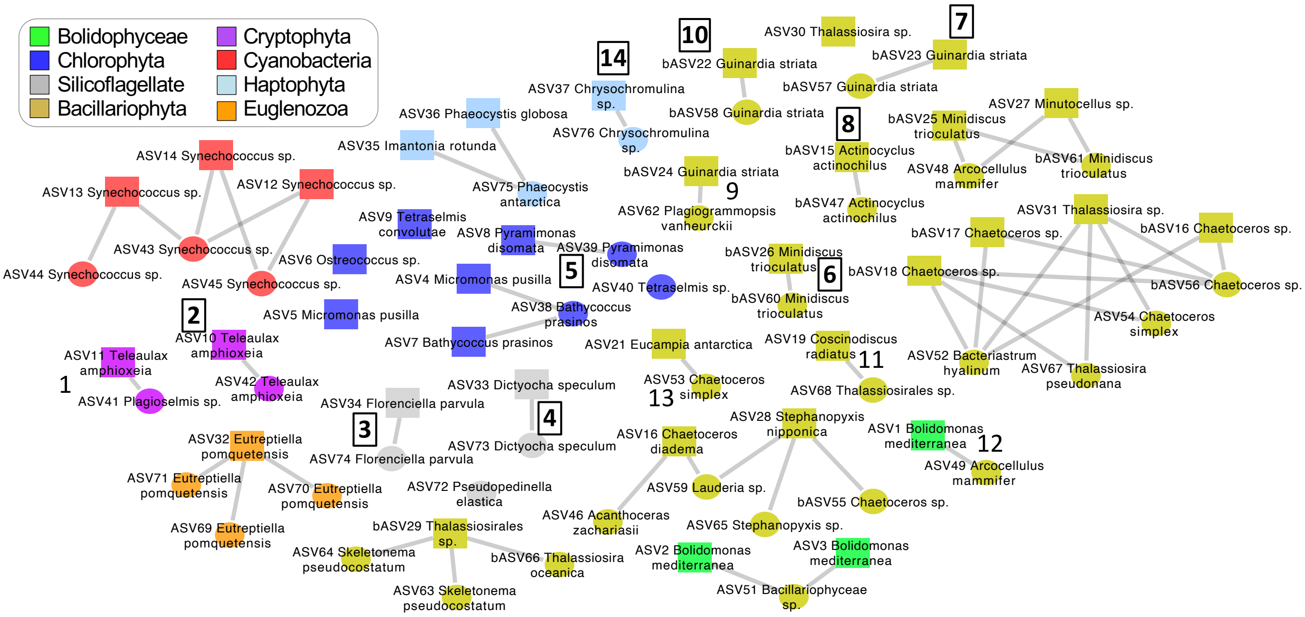

Fig S4. Network analysis of microbial associations for Top Twenty Bedford Basin ASVs between V4-V5 and V6-V8. The network shown is based on constructing an original network using all rarefied Bedford Basin samples (1, 5, 10, & 60m) and all ASVs and then reduced this larger network to only show statistically significant copresence relationships ($\alpha = 0.05$) for the top ASVs between V4-V5 (squares) and V6-V8 (circles). One-to-one relationships are numbered with boxes denoting equivalent taxonomic assignments. Only statistically significant positive correlations between V4-V5 and V6-V8 markers for each taxonomic group are shown.

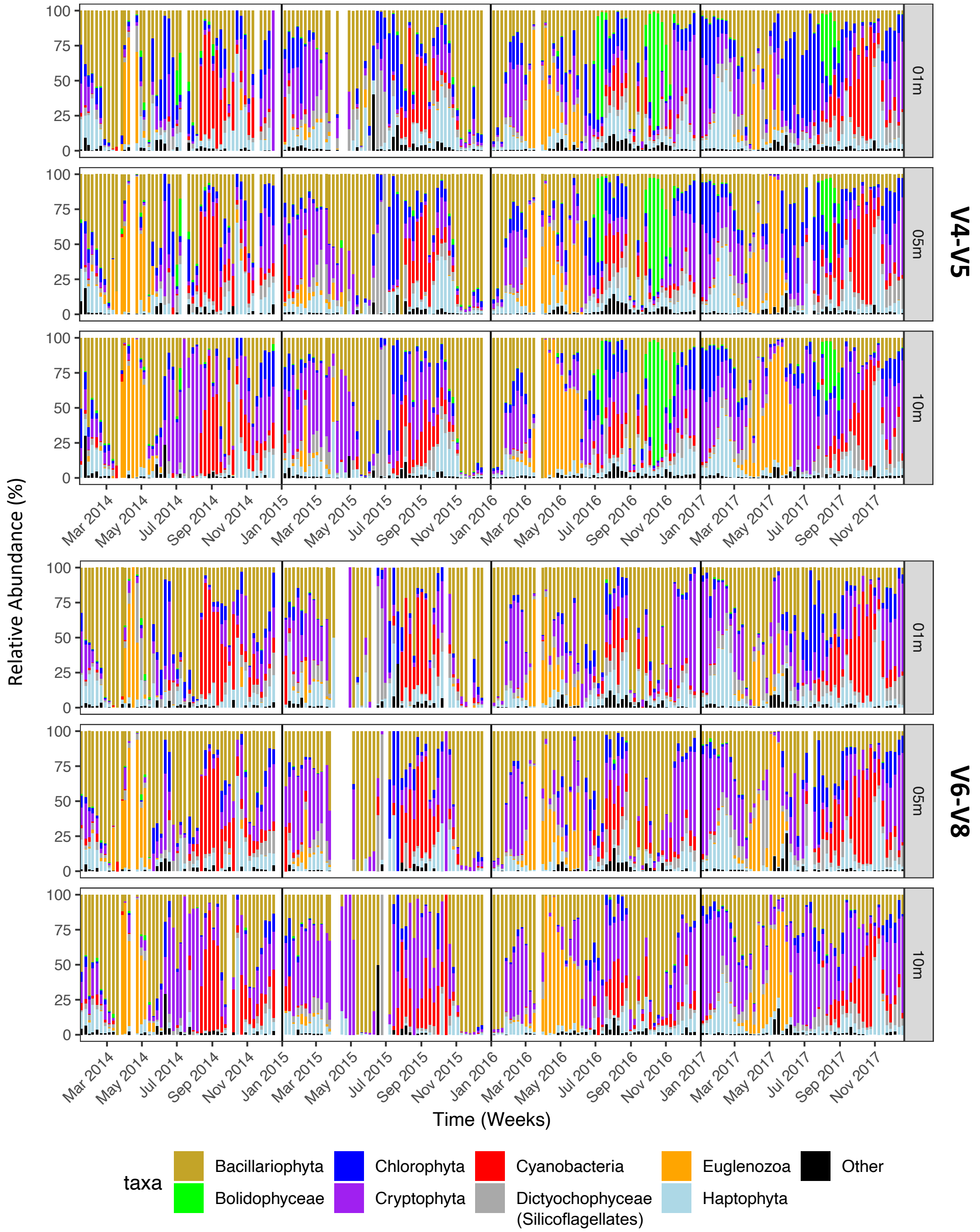

Fig S5. Weekly/seasonal phytoplankton trends in the Bedford Basin at 1, 5, & 10m depths as measured by unrarefied chloroplast and cyanobacterial *16S* relative abundances. Colors represent major phytoplankton groups. Note analysis uses all phytoplankton ASVs in dataset. Blank columns represent either missing samples, those with only bacterial *16S* reads, or those with unsuccessful sequencing (see Supplemental Data S5-S7).

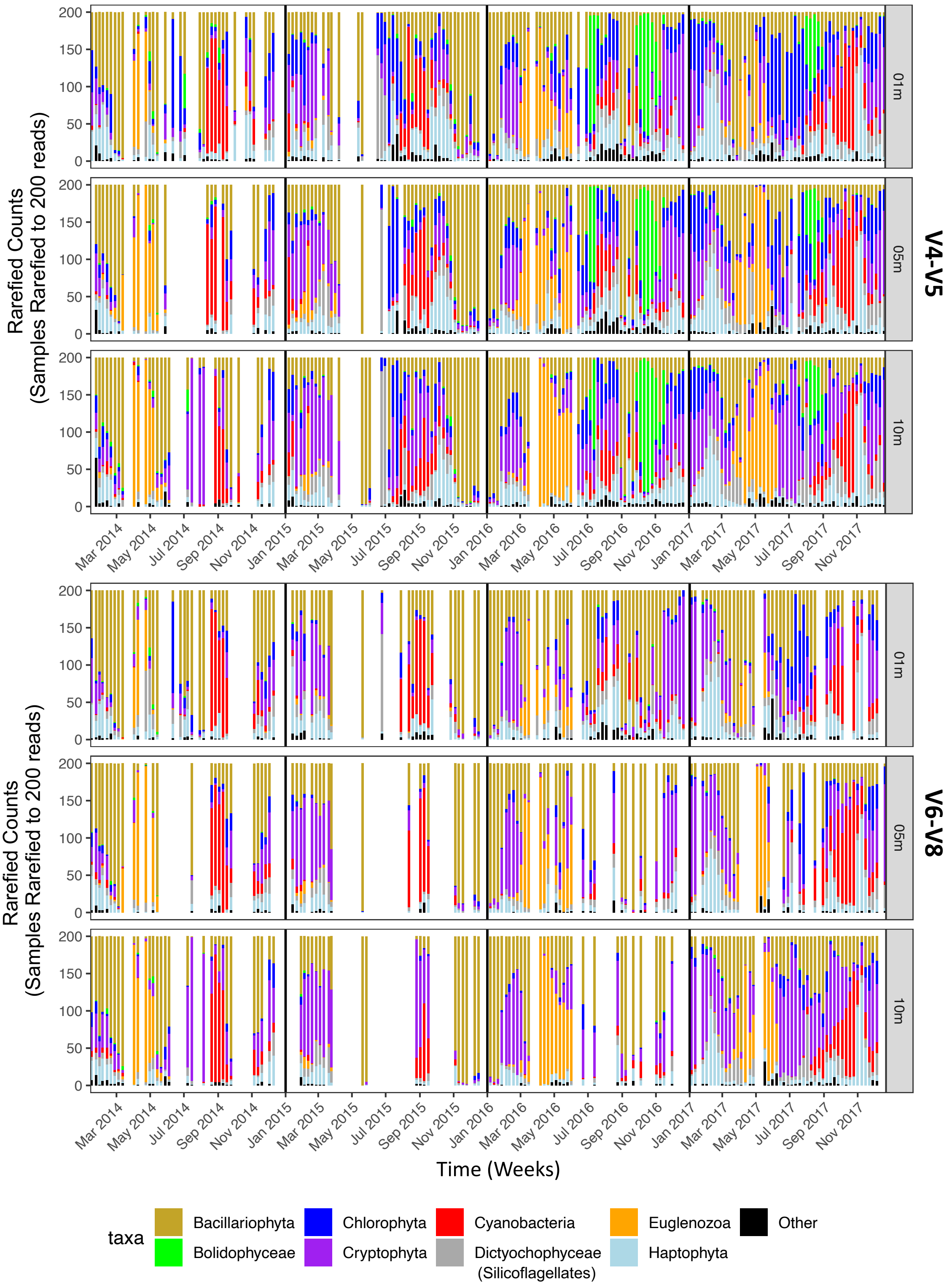

Fig S6. Rarefied abundances for all three surface depths (1,5, and 10m). Note that while various samples were removed during the rarefaction procedure, the full dataset does help to fill in any such gaps by capturing the diversity for at least one of the three surface depths (compare 1, 5, & 10m).

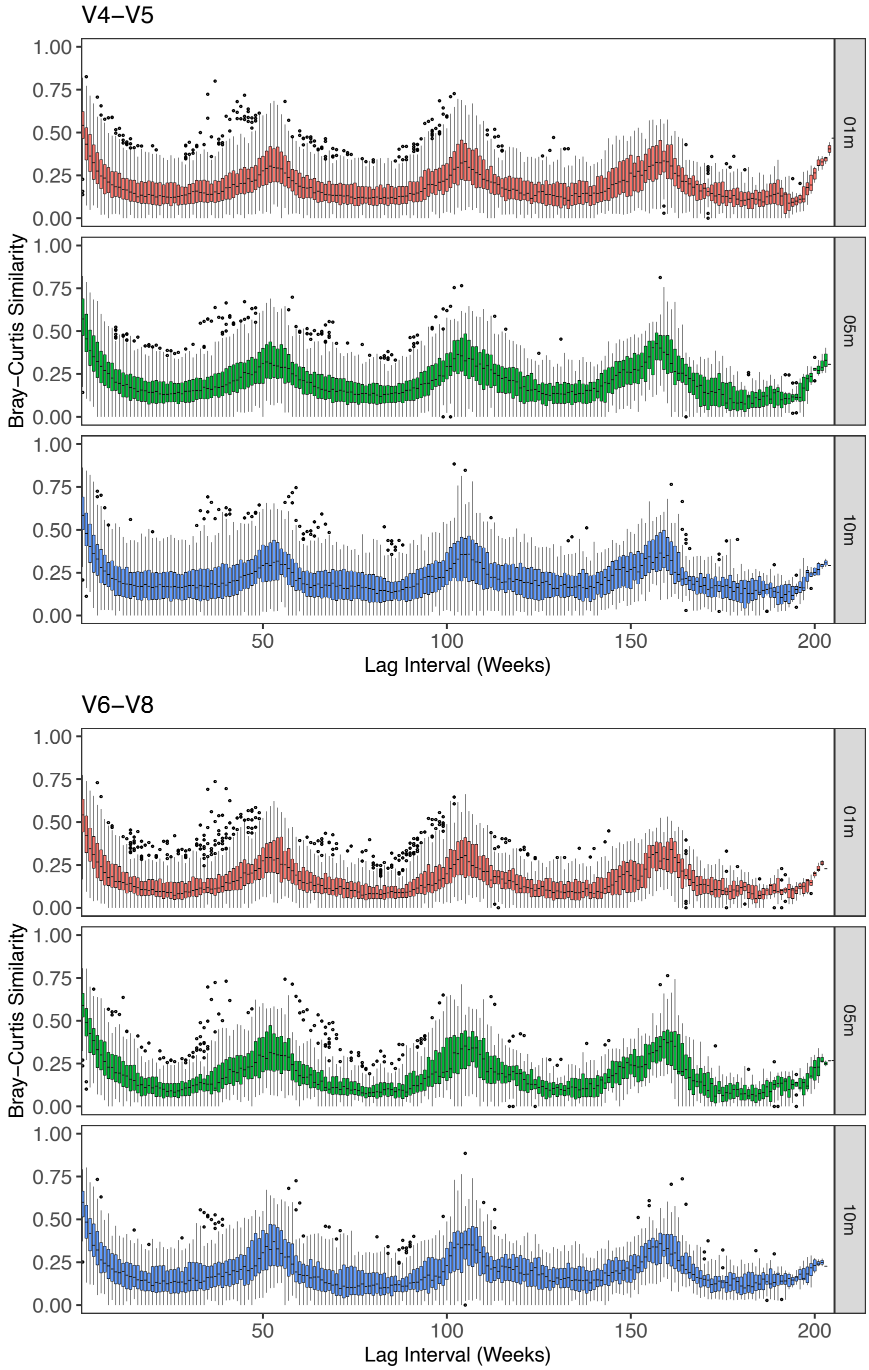

Fig S7. Analysis of Bray-Curtis Similarities between samples and then visualized according to the number of weeks between samples—data rarefied to 200 reads then converted to relative abundance & Hellinger transformed [47]. Major trend is that similarities peak at annual intervals. Compare to Fig S8 for unrarefied dataset.

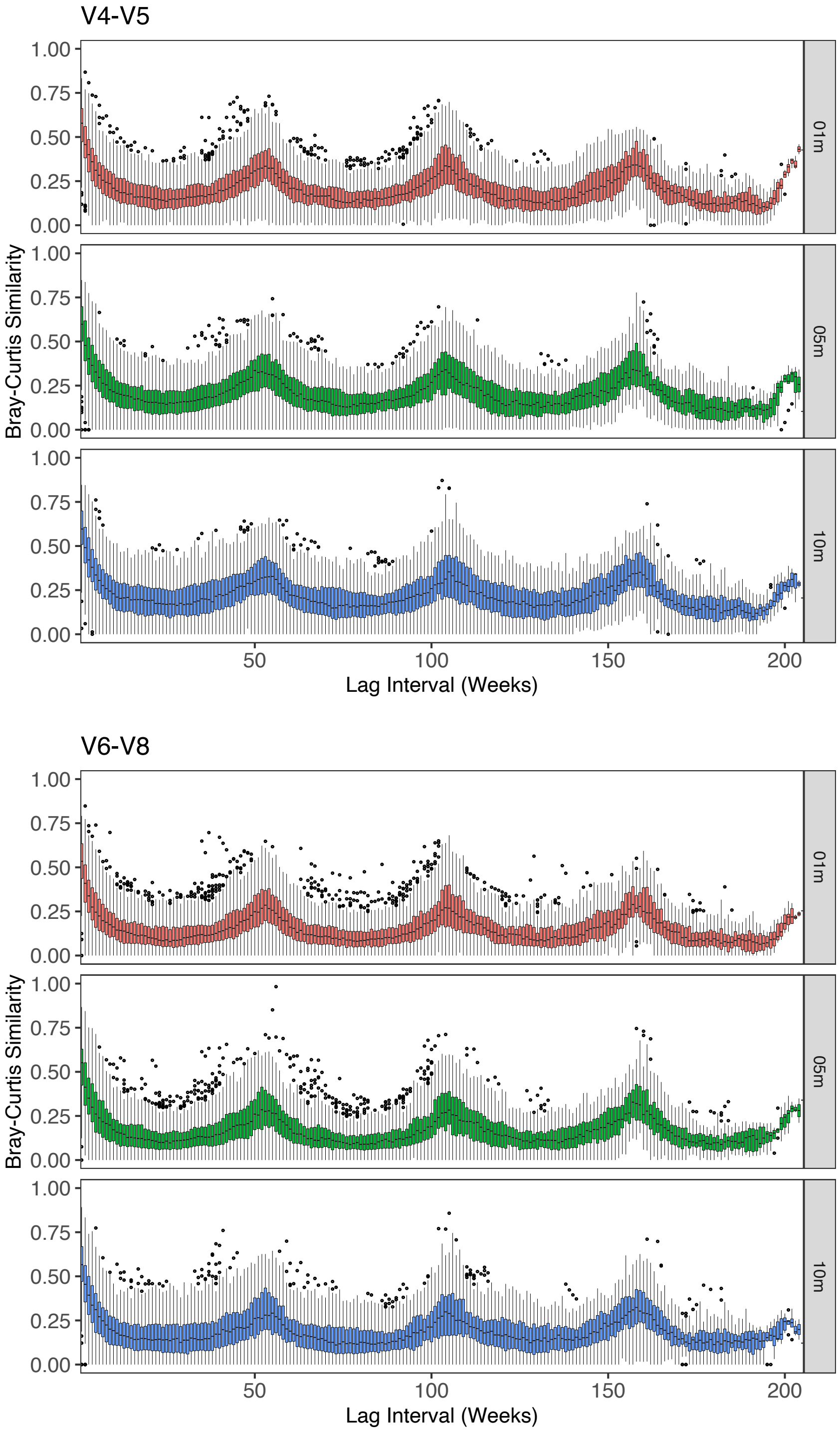

Fig S8. Analysis of Bray-Curtis Similarities between samples and then visualized according to the number of weeks between samples—data are unrarefied, converted to relative abundance, and Hellinger transformed [47]. Plot can be used to assess the effect of rarefaction on Fig S7.

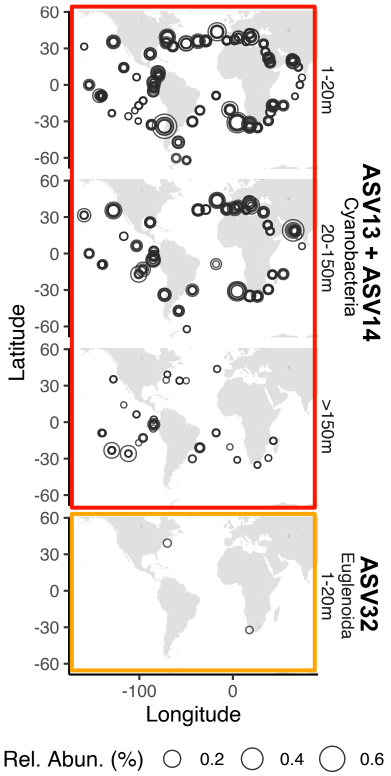

Fig S9. Oceanographic distributions (via *Tara* Oceans $_{mi}$TAGs) for cyanobacterial and Euglenoza indicator species that are present in the Bedford Basin during the fall and spring, respectively. Relative abundances & distributions are based on *Tara* Oceans data provided by [51,52]. Only *Tara* $_{mi}$TAGs with 100% pair-wise similarity and 100% coverage to our ASVs plotted.

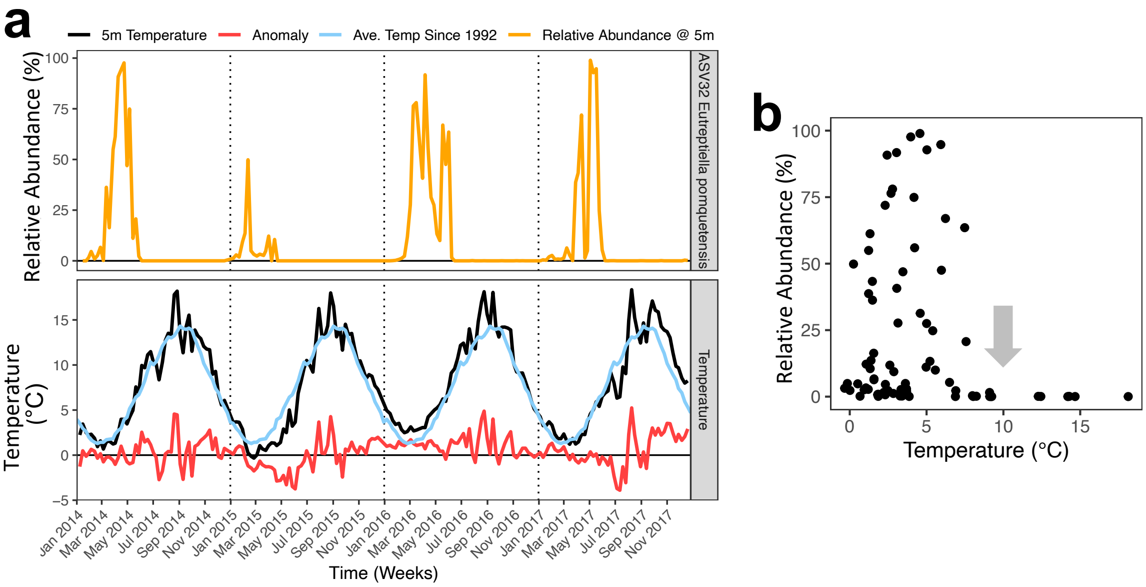

Fig S10. Relationship between temperature and *E. pomquetensis* as detected by the Bedford Basin molecular time-series. **(a)** Change in unrarefied relative abundance and temperature versus time; shows how sub-zero temperature in 2015 corresponded to decrease in relative abundance. **(b)** Unrarefied Relative abundance values versus temperature; shows how higher relative abundance were found generally above 0°C and less than 10°C (arrow).

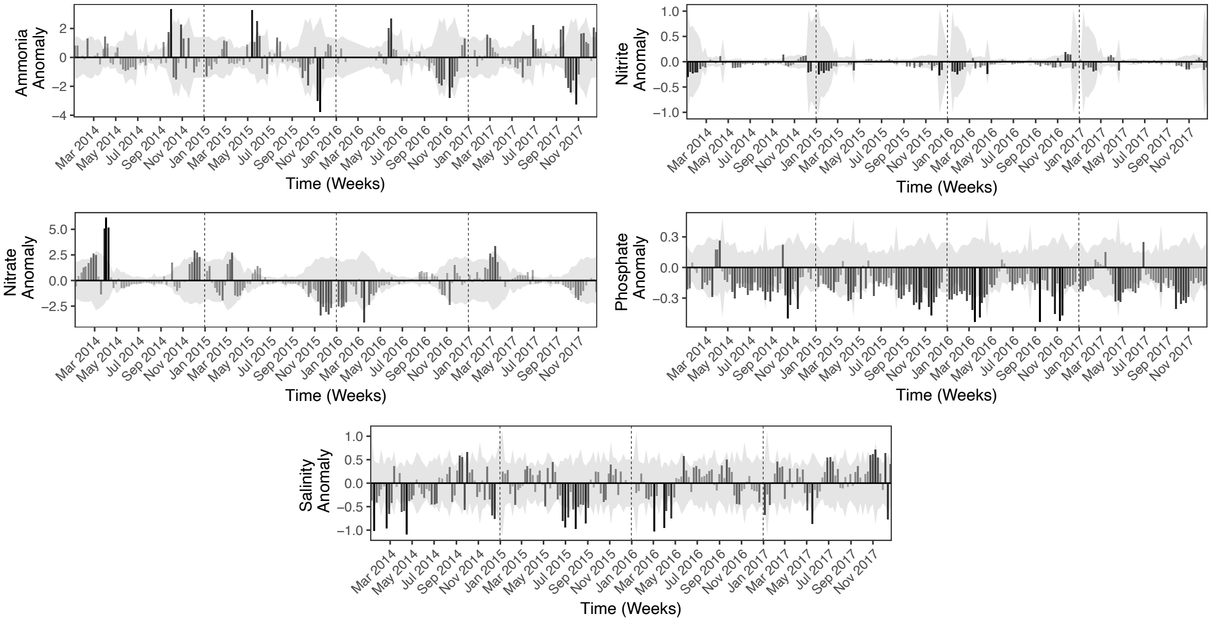

Fig S11. Salinity anomalies and nutrient anomalies. Values are the positive or negative difference relative to the 5m weekly mean for 1992–2017 (shading = standard deviations).

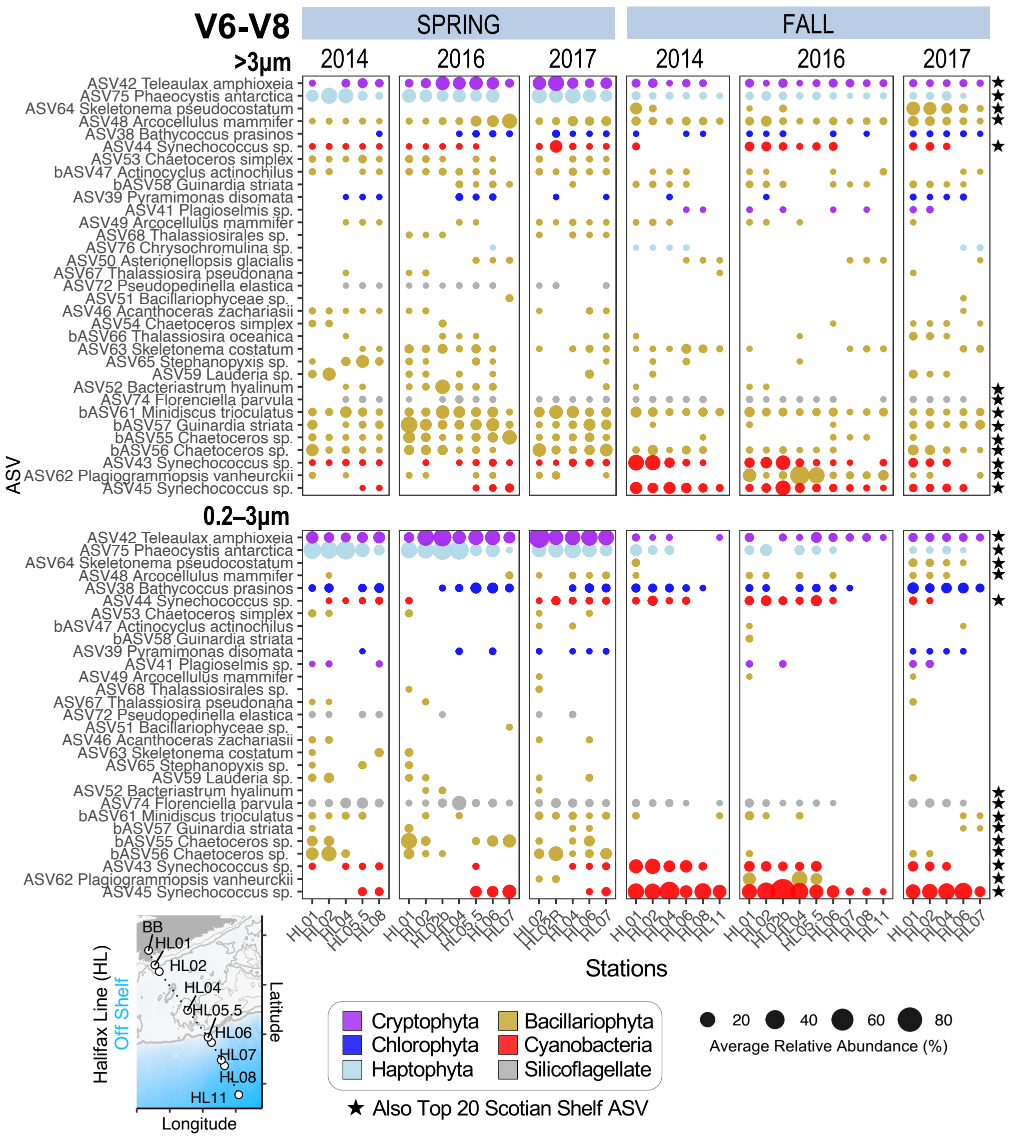

Fig S12. Top Twenty phytoplankton ASVs observed in the Bedford Basin time series are also detected seasonally in nearby Atlantic Zone Monitoring Program (AZMP) stations along the Halifax Line (HL). V6-V8 average relative abundances for individual ASVs throughout the water column for >3µm and 0.2–3µm fractions (top and bottom panels, respectively; data unrarefied). To help visualize presence/absence trends, ASVs are organized along the *y*-axis according to hierarchical clustering using the complete linkage method, a Euclidean distance matrix, and scaled total relative abundance per ASV. Starred ASVs (★) are sequence variants that also appear among the top twenty AZMP ASVs in each sample. Dominant ASVs from the Bedford Basin without a counterpart in the AZMP are not shown. The relative abundance calculation is the same as that reported in the caption of Fig 4.

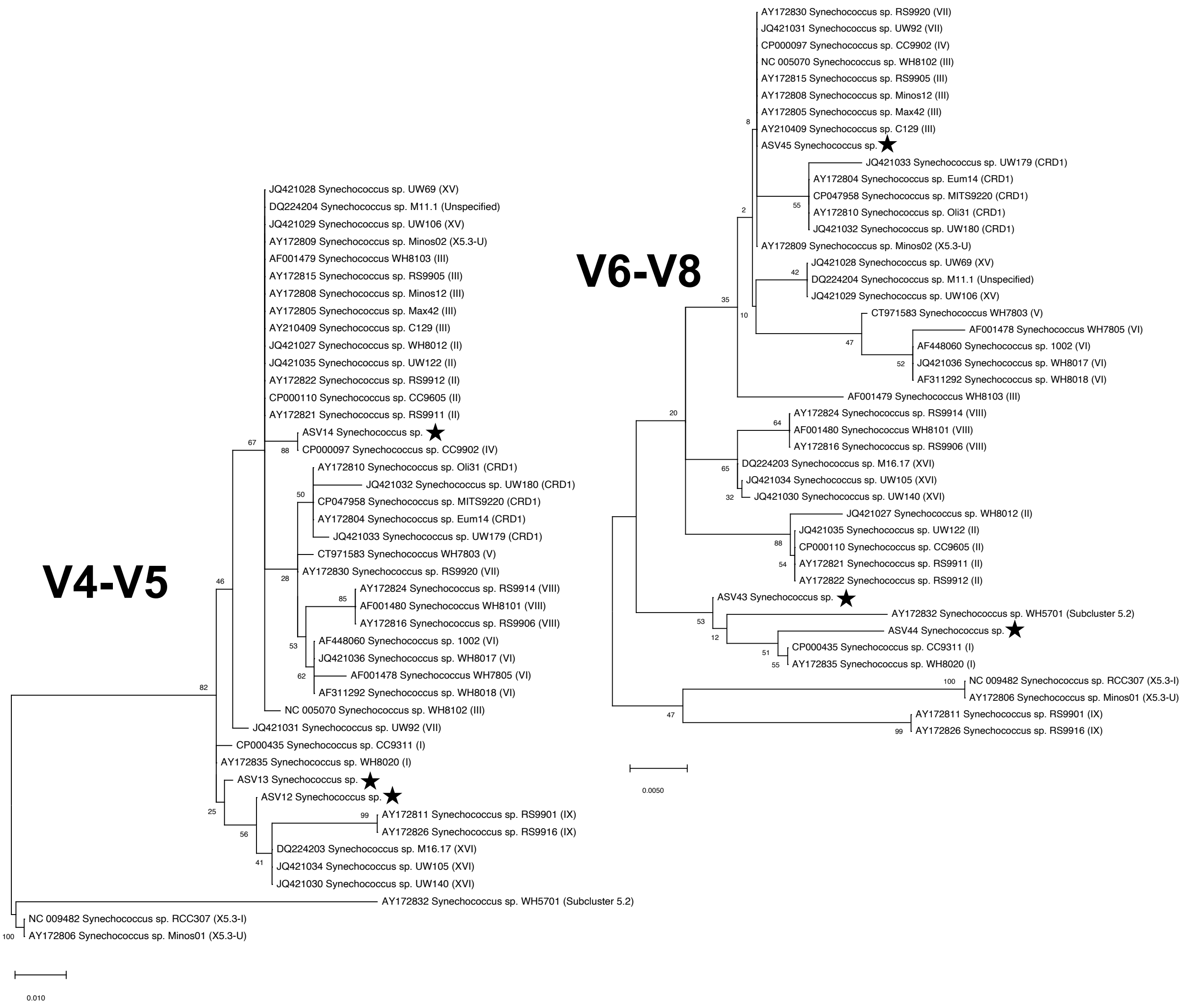

Fig S13. Phylogenetic assessment of *Synechococcus* ecotypes. For these trees we: aligned nucleotides via *MUSCLE* [45], trimmed sequences to either V4-V5 or V6-V8 ASV lengths, conducted a DNA substitution model test, assumed partial deletion at 95% to account for any missing sequence that remained after trimming, and set bootstrap replicates to 500 (all conducted within *MEGA* [46]. Figure also makes use of literature regarding known *Synechococcus* ecotypes [49,50].

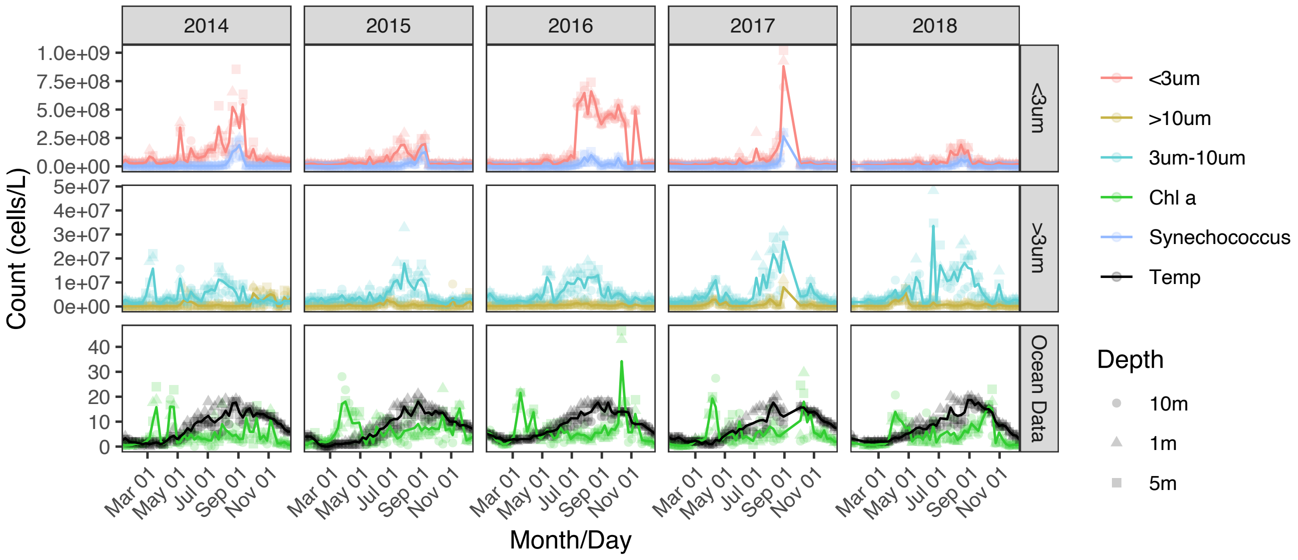

Fig S14. Additional time-series image of Bedford Basin flow cytometry. Figure shows all size-specific fractions and *Synechococcus*, as well as Ocean Data for chlorophyll *a* (Chl a) and Temperature. Lines represent averages of 1-10m data.

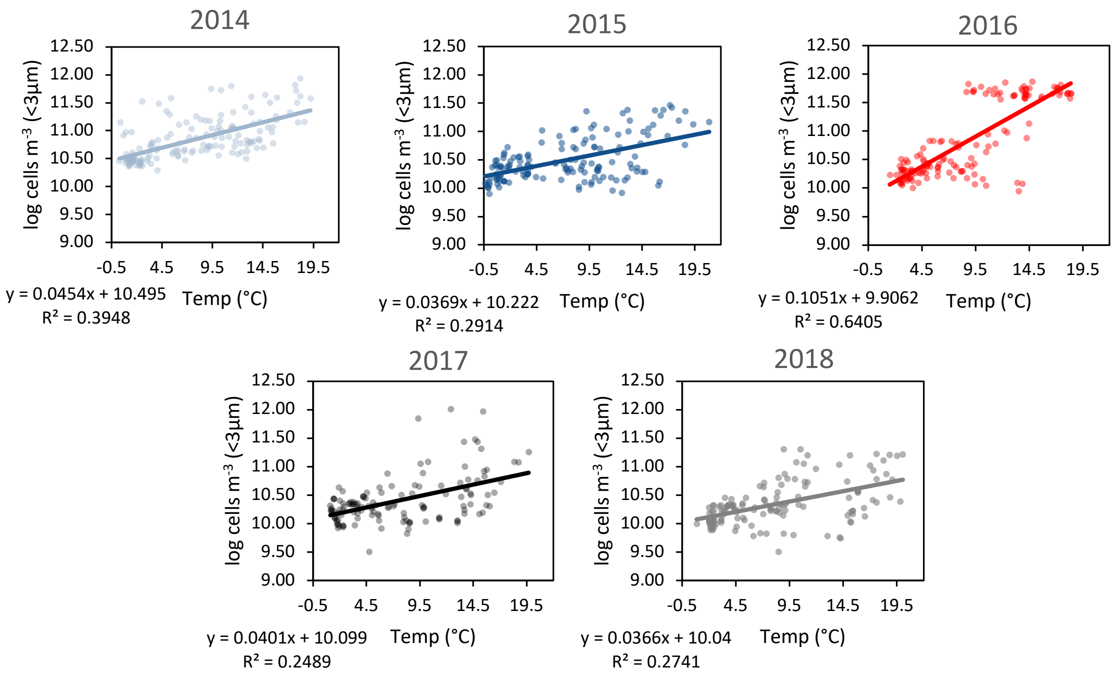

Fig S15. Relationship between <3µm cell densities and temperature separated into yearly plots. Trendlines plotted as linear relationships.
