# Supplementary material for: Highly-resolved interannual phytoplankton community dynamics of the coastal Northwest Atlantic
Source: ISME Commun. 2022 Apr 20;2:38. doi: 10.1038/s43705-022-00119-2 (PMC9723599; doi:10.1038/s43705-022-00119-2)
Supplement: Supplementary file 2 — Supplemental Text and Supplemental Tables [file 43705_2022_119_MOESM2_ESM.pdf]

SUPPLEMENTAL TEXT & SUPPLEMENTAL TABLES for

Highly resolved interannual phytoplankton community dynamics of the coastal Northwest Atlantic

Brent M. Robicheau, Jennifer Tolman, Erin M. Bertrand, Julie LaRoche

Supplemental Methods

Supplemental Methods S1. Flow-cytometry Gates: Flow cytometry gates for autofluorescence and size (forward scatter or FSC) were as in [9], and then also refined using both in-house axenic phytoplankton cultures with known autofluorescence/FSC profiles and Fluroesbrite microspheres (Polysciences, USA). An event exclusion threshold was set at <800 for Chlorophyll. Gates were as follows: Cryptophytes [high Chl, high PE, high FSC], Eukaryotes [high FSC, high Chl], Picoeukaryotes [low Chl, low PE, & lower FSC], and *Synechococcus* [low Chl, high PE, and low FSC] (Fig S1). Less than 3μm, 3–10μm, and >10μm flow cytometry gates were approximated using fluorescent microspheres at 0.7, 3, 6, and 10μm (Polysciences, USA; Spherotech, USA; BD, USA; Polysciences, USA, respectively).

Supplemental Methods S2. Amplicon Sequence Variants (ASVs): Raw read qualities were checked with *FASTQC* [94] and *MultiQC* [95]. Primer sequences were removed using *cutadapt* [96]. Paired-end reads were stitched together using *PEAR* [97], then imported into *QIIME2* [36]. Low-quality reads were removed, and the remaining reads denoised into ASVs using *deblur* [98]. As part of the user-specified *deblur* trim settings V6-V8 reads and V4-V5 reads were trimmed to 380bp and 350bp, respectively. ASVs with sequence read frequencies less than [0.001 × mean sample depth] were attributed to sequencer bleed-through and removed. Initial taxonomy was assigned via a Naïve-Bayes approach using the *classify-sklearn* command [36, 99] and a full-length *16S* trained classifier (based on SILVA database v132)[38].

Phytoplankton taxonomy was further refined with a *PhytoREF*-trained classifier [37] to reclassify any ASVs initially designated as either 'cyanobacteria' or 'chloroplast' by the SILVA database [38].

Supplemental Methods S3. R packages and Maps: The following packages were used for data visualizations/statistical analyses unless otherwise specifically indicated in the main text: *ggplot2* [48], *ggrepel* [100], *reshape2* [101], *scales* [102], and *cowplot* [103]. Any 'packages' mentioned within the text are also R packages [40]. Note that the *stat_summary* function in *ggplot2* [48] is used in various instances to plot the mean as a line across the time-series.

The data in Figure S12, were organized using hierarchical clustering via the *hclust* function in the *stats* package [40] and through the *ggdendro* [104] and the *scale* function in R [40].

Maps were generated using the *mapdata* [102], *ggrepel* [100], and *ggnewscale* [103] packages in R. Bathymetry data were retrieved from the online *ERDDAP* [104] server via its *griddap* protocol/data access form and the *ETOPO1* topography dataset [ID = *etopo180*] [ref. 105]; for this dataset the institution/creator is listed as the National Oceanic and Atmospheric Administration (NOAA) & the National Geophysical Data Center (NGDC).

Supplemental Methods S4. Comparison to Tara Oceans $_{mi}$TAGs for *Synechococcus* and Euglenozoa: For the distribution of cyanobacterial and Euglenozoa ASVs across the *TARA* Oceans samples, we retrieved metagenomic Illumina tag ($_{mi}$TAG) count data from the companion website to Sunagawa et al. [51] (see: ocean-microbiome.embl.de/companion.html; Last Accessed 15-Jun-2020), which also contains reference data for Logares et al. [52]. Our ASV sequences were locally aligned to the SILVA *16S* sequences from the *TARA* website above using

the online *BLAST* server [42,43], thus facilitating the retrieval of count data for $_{mi}$TAGs that shared similarity to our own ASVs [52]. Matches were limited to ASVs with 100% pair-wise identity (PI) and 100% coverage.

Supplemental Methods S5. V4-V5 versus V6-V8 Network Analysis: For the network, rarified data (all ASVs & all depths) were converted to relative abundance, and a minimum occurrence of 20 reads across all samples was required (removes rarer ASVs). Using the ensemble approach [106] the following methods were used to calculate V4-V5 ASV versus V6-V8 ASV network associations: Pearson and Spearman correlations, Mutual Information similarities, as well as Bray Curtis and Kullback-Leibler dissimilarities, with 1,000 top and bottom edges retained for each [106]. Initial *p*-values were calculated through permutation [100 iterations] and randomization via row shuffling, in addition, the renormalize parameter was selected and final *p*-values also incorporated bootstrapping [100 iterations] [ref. 106]. Brown's method was selected for merging *p*-values with a Benjamini-Hochberg multiple test correction setting [53, 106, 107]. The significance threshold was set at $\alpha = 0.05$. These settings reflect parameters provided by https://psbweb05.psb.ugent.be/conet/microbialnetworks/conet_new.php (last accessed 5-Aug-2020).

Supplemental Results

Supplemental Results S1. Network analysis comparing V4-V5 versus V6-V8: The sparsity of full-length cp*16S* reference sequences for uncultivated phytoplankton often precluded linking ASVs from the two variable regions to a common reference sequence. We conducted a network analysis to determine if any of the top ASVs could be directly correlated between the two *16S* markers using their temporal abundance profiles (Fig. S4). Fourteen one-to-one ASV

co-occurrences between the two *16S* markers were found. The majority (64.3%) of these had the same species name. Although symbiotic and/or mutualistic interactions cannot be excluded when interpreting the network, ASVs with significant network correlations yet different identification could have originated from the same taxon. One example is bASV24 *Guinardia striata* versus ASV62 *Plagiogrammopsis vanheurckii* (Fig. S4 and 3).

Supplemental Results S2. Trends for NMDS of closely related ASVs: NMDS plots indicate that for 2014–2017: (i) there were likely two ecotypes/strains of *Bolidomonas mediterranea* observed in the Bedford Basin [ASV1 associated with high salinity]; (ii) two ASVs of *Synechococcus* for each variable region that correlated with higher temperature [V4-V5 = ASV12 & 14; V6-V8 = ASV43] while another ASV was correlated with higher salinity [V4-V5 = ASV13; V6-V8 = ASV44 & 45]; (iii) the *Eutreptiella pomquetensis* ASVs observed in the V6-V8 dataset showed matching temporal distributions questioning the biological/ecological significance of the three ASVs; (iv) the detection of different ecotypes for several diatom species is suggested based on their preference for different environmental conditions, for example two ecotypes/strains of *Chaetoceros simplex* were detected by V6-V8 (with ASV53 somewhat associated with high salinity).

SUPPLEMENTAL DISCUSSION

Supplemental Discussion S1. Discordance between historical and new molecular data (this study): Amongst the predominant diatoms in our dataset were species from *Chaetoceros*, *Coscinodiscus*, *Eucampia*, *Pyramimonas*, *Skeletonema*, and *Thalassiosira*. Each of these genera have been reported in the Bedford Basin previously, with *Chaetoceros*, *Skeletonema*, and *Thalassiosira* consistently reported by multiple researchers [27, 28, 108–111].

While none of the species-level identifications from these studies matched those found through our *cp16S* analysis, it is possible that this discordance could have arisen from the use of molecular data (herein) versus morphological data (previous research) for species assignment. For example, although we identified *Skeletonema pseudocostatum* as the dominant *Skeletonema* species, *S. costatum* has been most often reported in the Bedford Basin [28, 110, 111]. Medlin et al. [112] propose that these two species can be readily distinguished morphologically, but the ability to do so depends on rather detailed knowledge of diatom morphological traits and potentially the use of electron microscopy (e.g. [113,114]). One should note, however, that there was indeed overlap for some species-level identifications; for instance, the Silicoflagellate, *Dictyocha speculum*, was the same species reported by Li et al. [58]. Taxonomic discordance may also arise from species under-representation in reference sequence datasets (discussed further below in Discussion S2); although alignment hits were >97% for *Chaetoceros*, *Skeletonema* and *Thalassiosira*, in several cases matches still had <100% PI (see Table S2).

One should also recognize that the study of Willis et al. [35] also recovered Bolidophyceae primarily by V4-V5, and that the results we presented in our study for a much larger dataset agrees with this earlier finding.

Supplemental Discussion S2. Coherence in the multiyear phytoplankton community composition: Our taxonomic identifications based on *cp16S* metabarcoding were generally consistent with previous microscopy records for larger phytoplankton, while providing a much more detailed taxonomic identification of smaller phytoplankton. It was evident that taxonomic discordance could have arisen due to differences in molecular data (herein) versus historical morphological data, for example, genera seemed to match well but species often did not (see Discussion S1); as mentioned above in Discussion S1, another possible source of discordance

may have been a lack of necessary reference sequences in GenBank, which would understandably hinder one's ability to arrive at a variant's truest taxonomy. For several of the dominant ASVs detected by both variable regions, similar reference accession codes were returned as top BLAST matches; this congruency may be due to the *nr* database [39] lacking sufficient sequences for the true species corresponding to each ASV, or may be a product of characterizing the same dominant microbial population regardless of marker choice (albeit with some differences in sequence conservation, as the pair-wise identity matches were not always 100%). More full length *cp16S* sequences are needed for type materials if researchers hope to further optimize the investigation of phytoplankton diversity via chloroplast/plastid subsets generated through *16S* metabarcoding. In future, large metabarcoding datasets of time series samples (such as the one presented herein) will also be useful in identifying and characterising novel cryptic species/strains that may currently lack the sampling frequency needed to discover their underlying ecologies (for instance, see [67]). For example, the taxonomic resolution afforded in this study allowed for the identification of closely related ecotypes from the same species that thrive under different environmental conditions (Fig. 3b).

In general, there was also a lack of phototropic dinoflagellates observed in our study and this is mostly likely explained by low cell densities for this group. We did observe four Dinophyta ASVs belonging to *Karlodinium veneficum* (for V4-V5 at 0.002% of entire dataset) and *Karenia mikimotoi* (for V4-V5 at 0.024% of dataset, and for V6-V8 at 0.005% of dataset) at very low relative abundances. It is possible that some of the dinoflagellates in the Bedford Basin may have been non-photosynthetic (i.e., lacking chloroplasts) [115], although Dasilva et al. [69] have shown through *18S* rRNA cloning that many of the dinoflagellates identified in April and October 2009 along the nearby Scotian Shelf were mixotrophic. DNA pre-filtration would have also theoretically allowed for the capture of dinoflagellates given that Lehman [28] reported an

average BB dinoflagellate cell volume of 54,444 ± 3,120 μm^3; for one of the larger dinoflagellates they identify, *Dinophysis norvegica*, its size is upwards of 70µm in one dimension [116]. Given that metabolism and cell size cannot explain low dinoflagellate detection, we turn instead to cell densities. Historical microscopy observations show that annual average ratios in this region are on the order of 1055:10:1 for *Synechococcus* : Diatoms : Dinoflagellates [5]. Based on these ratios, there exists a relatively low expected probability of recovering dinoflagellate cpDNA gene sequences compared to diatoms in this fjord, hence, low dinoflagellate concentrations seem the most plausible explanation for dinoflagellate rarity in our molecular data.

Supplemental Discussion S3. *Eutreptiella* and historical reporting: Reasons for low *Eutreptiella* reporting in the past may including selective exclusion during flow cytometry, water sample treatment, and morphology scoring. Unlike *Synechococcus*, which has an average cell size of 0.9µm [117], *E. pomquetensis* (previously *Tetreutreptia pomquetensis*) has an average cell length of ≥70µm [61] and would be selectively excluded by pre-filtration of flow cytometry samples. McLachlan et al. [61] also indicate that unless fixed in glutaraldehyde or Lugol's solution, *E. pomquetensis* is very sensitive to increased temperatures, with exposure to ≥10°C generally lethal. Thus, one can assume that unless Bedford Basin water samples are rapidly fixed at near *in situ* temperatures, there is a likelihood of biasing microscopy samples against *E. pomquetensis*. Furthermore, microscopic identification could have misclassified *E. pomquetensis* cells or placed them into a broader taxonomic group (e.g., flagellate). Intriguingly, another Euglenozoa, *Euglena* sp., is listed as occurring in the Bedford Basin during the 1990s [58].

References appearing only in Supplemental Information

94. Andrews S. FastQC: A Quality Control Tool for High Throughput Sequence Data. 2010. http://www.bioinformatics.babraham.ac.uk/projects/fastqc/
95. Ewels P, Magnusson M, Lundin S, Käller M. MultiQC: summarize analysis results for multiple tools and samples in a single report. *Bioinformatics* 2016; **32**: 3047–3048.
96. Martin M. Cutadapt removes adapter sequences from high-throughput sequencing reads. *EMBnet.journal* 2011; **17**: 10–12.
97. Zhang J, Kobert K, Flouri T, Stamatakis A. PEAR: a fast and accurate Illumina Paired-End reAd mergeR. *Bioinformatics* 2014; **30**: 614–620.
98. Amir A, McDonald D, Navas-Molina JA, Kopylova E, Morton JT, Zech Xu Z, et al. Deblur Rapidly Resolves Single-Nucleotide Community Sequence Patterns. *mSystems* 2017; **2**: e00191-16.
99. Pedregosa F, Varoquaux G, Gramfort A, Michel V, Thirion B, Grisel O, et al. Scikit-learn: Machine learning in Python. *the Journal of machine Learning research* 2011; **12**: 2825–2830.
100. Slowikowski K. ggrepel: Automatically Position Non-Overlapping Text Labels with 'ggplot2'. R package version 0.8.2. 2020. https://CRAN.R-project.org/package=ggrepel
101. Wickham H. Reshaping data with the reshape package. *Journal of statistical software* 2007; **21**: 1–20.
102. Wickham H, Seidel D. scales: Scale Functions for Visualization. R package version 1.1.1. 2020. https://CRAN.R-project.org/package=scales
103. Wilke C. cowplot: Streamlined Plot Theme and Plot Annotations for 'ggplot2'. R package version 1.0.0. 2019. https://CRAN.R-project.org/package=cowplot
104. de Vries A, Ripley B. ggdendro: Create Dendrograms and Tree Diagrams Using

‘ggplot2’. R package version 0.1-20. 2016. https://CRAN.R-project.org/package=ggdendro

102. Brownrigg R. mapdata: Extra Map Databases, R package version 2.3.0, Original S code by RA Becker & AR Wilks. 2018. https://CRAN.R-project.org/package=mapdata

103. Campitelli E. ggnewscale: Multiple Fill and Colour Scales in ‘ggplot2’, R package version 0.4.1. 2020. https://CRAN.R-project.org/package=ggnewscale

104. Simons RA. ERDDAP. 2019. NOAA/NMFS/SWFSC/ERD, Monterey, CA. https://coastwatch.pfeg.noaa.gov/erddap

105. Amante C, Eakins B. ETOPO1 1 Arc-Minute Global Relief Model: Procedures, Data Sources and Analysis. *NOAA Technical Memorandum NESDIS NGDC-24*. 2009. National Geophysical Data Center, NOAA. doi:10.7289/V5C8276M

106. Faust K, Sathirapongsasuti JF, Izard J, Segata N, Gevers D, Raes J, et al. Microbial Co-occurrence Relationships in the Human Microbiome. *PLOS Computational Biology* 2012; **8**: e1002606.

107. Brown MB. 400: A Method for Combining Non-Independent, One-Sided Tests of Significance. *Biometrics* 1975; **31**: 987–992.

108. Kepkay PE, Niven SEH, Jellett JF. Colloidal organic carbon and phytoplankton speciation during a coastal bloom. *J Plankton Res* 1997; **19**: 369–389.

109. Kranck K, Milligan TG. Macroflocs from diatoms: in situ photography of particles in Bedford Basin, Nova Scotia. *Marine Ecology Progress Series* 1988; **44**: 183–189.

110. Mayzaud P, Taguchi S. Spectral and Biochemical Characteristics of the Particulate Matter in Bedford Basin. *J Fish Res Bd Can* 1979; **36**: 211–218.

111. Conover RJ, Mayzaud P. Utilization of Phytoplankton by Zooplankton during the Spring Bloom in a Nova Scotia Inlet. *Can J Fish Aquat Sci* 1984; **41**: 232–244.

112. Medlin LK, Elwood HJ, Stickel S, Sogin ML. Morphological and Genetic Variation Within the Diatom *Skeletonema costatum* (bacillariophyta): Evidence for a New Species, *Skeletonema pseudocostatum*. *Journal of Phycology* 1991; 27: 514–524.

113. Kooistra WHCF, Sarno D, Balzano S, Gu H, Andersen RA, Zingone A. Global Diversity and Biogeography of *Skeletonema* Species (Bacillariophyta). *Protist* 2008; **159**: 177–193.

114. Sarno D, Kooistra W, Medlin L, Percopo I, Zingone A. Diversity in the genus *Skeletonema* (Bacillariophyceae). II. An assessment of the taxonomy of *S. costatum*-like species with the description of four new species. *Journal of Phycology* 2005; **41**: 151–176.

115. Schnepf E, ElbräChter M. Dinophyte chloroplasts and phylogeny - A review. *Grana* 1999; **38**: 81–97.

116. Carpenter EJ, Janson S, Boje R, Pollehne F, Chang J. The dinoflagellate *Dinophysis norvegica*: biological and ecological observations in the Baltic Sea. *European Journal of Phycology* 1995; **30**: 1–9.

117. Morel A, Ahn Y-H, Partensky F, Vaulot D, Claustre H. *Prochlorococcus* and *Synechococcus*: A comparative study of their optical properties in relation to their size and pigmentation. *Journal of Marine Research* 1993; **51**: 617–649.

118. Tsuchiya M, Chikaraishi Y, Nomaki H, Sasaki Y, Tame A, Uematsu K, et al. Compound-specific isotope analysis of benthic foraminifer amino acids suggests microhabitat variability in rocky-shore environments. *Ecology and Evolution* 2018; **8**: 8380–8395.

119. Tsuchiya M, Toyofuku T, Uematsu K, Brüchert V, Collen J, Yamamoto H, et al. Cytologic and Genetic Characteristics of Endobiotic Bacteria and Kleptoplasts of *Virgulinella fragilis* (Foraminifera). *Journal of Eukaryotic Microbiology* 2015; **62**: 454–469.

Supplemental Tables

Table S1. Output of indicator species test. 1–10m depths and all weeks used in analysis; data were rarified and converted to percent relative abundance. Significance codes: 0 '***', 0.001 '**', 0.01 '*' 0.05 '.'

V4-V5		
Association function: r.g		
Significance level (alpha): 0.05		
Total number of species: 37		
Selected number of species: 36		
Number of species associated to 1 group: 22		
Number of species associated to 2 groups: 14		
Number of species associated to 3 groups: 0		
**List of species associated to each season(s)**	**Stat**	**p-value**
**Group Fall #sps. 5**		
ASV12.Synechococcus.sp.	0.504	1e-04 ***
ASV11.Teleaulax.amphioxeia	0.443	1e-04 ***
ASV14.Synechococcus.sp..CC9902	0.387	1e-04 ***
ASV2.Bolidomonas.mediterranea	0.256	1e-04 ***
bASV22.Guinardia.striata	0.212	1e-04 ***
**Group Spring #sps. 6**		
ASV36.Phaeocystis.globosa	0.412	1e-04 ***
bASV17.Chaetoceros.sp.	0.39	1e-04 ***
bASV18.Chaetoceros.sp.	0.316	1e-04 ***
ASV31.Thalassiosira.sp.	0.311	1e-04 ***
ASV28.Stephanopyxis.nipponica	0.241	1e-04 ***
ASV21.Eucampia.antarctica	0.173	3e-04 ***
**Group Summer #sps. 4**		
ASV5.Micromonas.pusilla	0.473	0.0001 ***
ASV27.Minutocellus.sp.	0.304	0.0001 ***
ASV6.Ostreococcus.sp.	0.29	0.0001 ***
ASV9.Tetraselmis.convolutae	0.126	0.0125 *
**Group Winter #sps. 7**		
bASV24.Guinardia.striata	0.311	1e-04 ***
ASV8.Pyramimonas.disomata	0.295	1e-04 ***
ASV35.Imantonia.rotunda	0.261	1e-04 ***
bASV15.Actinocyclus.actinochilus	0.252	1e-04 ***
ASV19.Coscinodiscus.radiatus	0.222	1e-04 ***
bASV23.Guinardia.striata	0.206	1e-04 ***
ASV37.Chrysochromulina.sp.	0.193	1e-04 ***
**Group Fall+Summer #sps. 4**		
ASV13.Synechococcus.sp.	0.228	2e-04 ***
bASV26.Minidiscus.trioculatus	0.222	1e-04 ***
ASV1.Bolidomonas.mediterranea	0.201	3e-04 ***
bASV29.Thalassiosirales.sp.	0.173	3e-04 ***
**Group Fall+Winter #sps. 2**		
ASV30.Thalassiosira.sp.	0.272	1e-04 ***
ASV3.Bolidomonas.mediterranea	0.175	7e-04 ***
**Group Spring+Summer #sps. 4**		
ASV32.Eutreptiella.pomquetensis	0.4	0.0001 ***
bASV25.Minidiscus.trioculatus	0.256	0.0001 ***
bASV16.Chaetoceros.sp.	0.209	0.0002 ***
ASV16.Chaetoceros.diadema	0.159	0.0026 **
**Group Spring+Winter #sps. 2**		
ASV10.Teleaulax.amphioxeia	0.244	1e-04 ***
ASV34.Florenciella.parvula	0.233	1e-04 ***
**Group Summer+Winter #sps. 2**		
ASV7.Bathycoccus.prasinos	0.216	2e-04 ***
ASV4.Micromonas.pusilla	0.208	1e-04 ***

V6-V8		
Association function: r.g		
Significance level (alpha): 0.05		
Total number of species: 39		
Selected number of species: 37		
Number of species associated to 1 group: 24		
Number of species associated to 2 groups: 13		
Number of species associated to 3 groups: 0		
**List of species associated to each season(s)**	**Stat**	**p-value**
**Group Fall #sps. 5**		
ASV43.Synechococcus.sp.	0.587	1e-04 ***
ASV41.Plagioselmis.sp.	0.466	1e-04 ***
ASV45.Synechococcus.sp.	0.409	1e-04 ***
ASV64.Skeletonema.pseudocostatum	0.294	1e-04 ***
ASV50.Asterionellopsis.glacialis	0.227	2e-04 ***
**Group Spring #sps. 9**		
bASV56.Chaetoceros.sp.	0.443	0.0001 ***
ASV75.Phaeocystis.antarctica	0.417	0.0001 ***
ASV59.Lauderia.sp.	0.349	0.0001 ***
ASV54.Chaetoceros.simplex	0.344	0.0001 ***
ASV67.Thalassiosira.pseudonana	0.316	0.0001 ***
bASV55.Chaetoceros.sp.	0.316	0.0001 ***
ASV52.Bacteriastrum.hyalinum	0.307	0.0001 ***
ASV65.Stephanopyxis.sp.	0.247	0.0001 ***
ASV53.Chaetoceros.simplex	0.166	0.0063 **
**Group Summer #sps. 3**		
ASV48.Arcocellulus.mammifer	0.367	1e-04 ***
bASV66.Thalassiosira.oceanica	0.221	1e-04 ***
ASV40.Tetraselmis.sp.	0.171	3e-04 ***
**Group Winter #sps. 7**		
ASV38.Bathycoccus.prasinos	0.349	1e-04 ***
ASV39.Pyramimonas.disomata	0.297	1e-04 ***
bASV57.Guinardia.striata	0.257	1e-04 ***
ASV68.Thalassiosirales.sp.	0.251	1e-04 ***
bASV47.Actinocyclus.actinochilus	0.234	1e-04 ***
ASV51.Bacillariophyceae.sp.	0.208	2e-04 ***
ASV76.Chrysochromulina.sp.	0.195	6e-04 ***
**Group Fall+Summer #sps. 4**		
ASV49.Arcocellulus.mammifer	0.224	0.0002 ***
bASV60.Minidiscus.trioculatus	0.22	0.0001 ***
bASV58.Guinardia.striata	0.188	0.0015 **
ASV44.Synechococcus.sp.	0.185	0.0016 **
**Group Fall+Winter #sps. 1**		
ASV62.Plagiogrammopsis.vanheurckii	0.31	1e-04 ***
**Group Spring+Summer #sps. 6**		
ASV71.Eutreptiella.pomquetensis	0.379	0.0001 ***
ASV69.Eutreptiella.pomquetensis	0.371	0.0001 ***
ASV70.Eutreptiella.pomquetensis	0.349	0.0001 ***
bASV61.Minidiscus.trioculatus	0.291	0.0001 ***
ASV72.Pseudopedinella.elastica	0.232	0.0001 ***
ASV46.Acanthoceras.zachariasii	0.167	0.0064 **
**Group Spring+Winter #sps. 2**		
ASV74.Florenciella.parvula	0.269	1e-04 ***
ASV42.Teleaulax.amphioxeia	0.233	2e-04 ***

Table S2. List of dominant ASVs that were manually identified to genus/species level. Also provided are reference accessions and *BLAST* pairwise-identities (PI) to the NCBI nucleotide (*nr*/*nt*) collection [39,42,43]. Note that when multiple reference accessions were found to match at equivalent PI and query coverage only a few are given as example. Please see methods section for further details on the approach used for resolving *BLAST* matches. NCBI sequences likely of endobiont origin were excluded during taxonomy assessment (e.g. sequences in [118,119]).

Taxon	Accessions (PI % | Query Coverage %)
ASV46 *Acanthoceras zachariasii*	NC_038009.1 (97.11 | 100)
bASV15 *Actinocyclus actinochilus*	FJ002163.1* (100 | 100)
bASV47 *Actinocyclus actinochilus*	FJ002163.1* (99.21|100)
ASV48 *Arcocellulus mammifer*	FJ002193.1 (100|100)
ASV49 *Arcocellulus mammifer*	FJ002193.1 (98.43|100)
ASV50 *Asterionellopsis glacialis*	FJ002233.1 (98.68|100)
ASV51 Bacillariophyceae sp.	FJ002233.1, AF514850.1 (98.68|100)
ASV52 *Bacteriastrum hyalinum*	FJ002166.1 (97.89|100)
ASV7 *Bathycoccus prasinos*	LN735275.2, FO082259.2 (100 | 100)
ASV38 *Bathycoccus prasinos*	FN563099.1 (100|100)
ASV1 *Bolidomonas mediterranea*	LN735367.3, AY702144.1 (98.29 | 100)
ASV2 *Bolidomonas mediterranea*	KC509524.1, LN735367.3, AY702144.1 (98.29-98.57 | 100) [May be *Leptocylindrus danicus,* however only 1bp more similar than other *Bolidomonas* and PhytoREF also suggests *Bolidomonas*]
ASV3 *Bolidomonas mediterranea*	LN735367.3, AY702144.1 (98.6 | 100)
ASV16 *Chaetoceros diadema*	MH011755.1, LN735283.2 (99.14 | 100)
ASV53 *Chaetoceros simplex*	KJ958479.1 (99.21|100)
ASV54 *Chaetoceros simplex*	LC088209.1, KJ958479.1 (99.21-99.74|100)
bASV17 *Chaetoceros* sp.	MH011755.1, LN735388.3, LN735283.2, AJ319825.1 (99.43 | 100)
bASV18 *Chaetoceros* sp.	LN735300.2 (100 | 100)
bASV16 *Chaetoceros* sp.	MH011753.1, JN207225.1 (99.43-99.71 | 99-100)
bASV55 *Chaetoceros* sp.	LC088209.1, FJ002204.1 (99.47-100|100)
bASV56 *Chaetoceros* sp.	NC_053621.1, FJ002215.1, FJ159135.1 (98.42|100)
ASV37 *Chrysochromulina* sp.	LN735342.3, LN735328.3, LN735326.3, AB196967.1 (99.71 | 100) – Matches several homotypic synonyms for *Chrysochromulina* genus
ASV76 *Chrysochromulina* sp.	AB196966.1 (98.42|100)
ASV19 *Coscinodiscus radiatus*	AJ536462.1 (98.86 | 100), possibly *C. granii* (new sequence Apr-2021 MW561225.1 (99.14 | 100))
ASV33 *Dictyocha speculum*	NC_043929.1* (100 | 100)
ASV73 *Dictyocha speculum*	NC_043929.1* (99.74|100)
ASV21 *Eucampia antarctica*	FJ002159.1 (99.71 | 100)
ASV32 *Eutreptiella pomquetensis*	KY706202.1*, EU750699.1 (100 | 100)
ASV69 *Eutreptiella pomquetensis*	KY706202.1* (99.47|100)
ASV70 *Eutreptiella pomquetensis*	KY706202.1* (99.74|100)
ASV71 *Eutreptiella pomquetensis*	KY706202.1* (100|100)
ASV34 *Florenciella parvula*	NC_044407.1*, LN735277.2 (100 | 100)
ASV74 *Florenciella parvula*	NC_044407.1* (100|100)
bASV22 *Guinardia striata*	LN735412.3, NC_037998.1* (99.42-98.85 | 99-100)
bASV23 *Guinardia striata*	NC_037998.1* (99.43 | 100)
bASV24 *Guinardia striata*	NC_037998.1* (99.43 | 100)
bASV57 *Guinardia striata*	NC_037998.1* (98.95|100)
bASV58 *Guinardia striata*	NC_037998.1* (98.16|100)
ASV35 *Imantonia rotunda*	LN735489.3, AY702150.1 (100 | 100) – new species name is *Dicrateria rotunda*
ASV59 *Lauderia* sp.	FJ002202.1, AJ536459.1 (100|100)
ASV4 *Micromonas pusilla*	LN735276.2, MT136879.1, EF051748.1 (100 | 100)
ASV5 *Micromonas pusilla*	LN735344.3 (99.14 | 100)
bASV26 *Minidiscus trioculatus*	FJ002231.1* (100 | 100)
bASV25 *Minidiscus trioculatus*	FJ002231.1* (99.71 | 100)
bASV60 *Minidiscus trioculatus*	FJ002231.1* (99.74|100)
bASV61 *Minidiscus trioculatus*	FJ002231.1* (100|100)
ASV27 *Minutocellus* sp.	LN735501.3, FJ002193.1, FJ002199.1 (99.15-99.43|100) – was also similar to *Arcocellulus mammifer*
ASV6 *Ostreococcus* sp.	LN735433.3, LN735218.2, AY702141.1 (100 | 100)
ASV75 *Phaeocystis antarctica*	JN117275.2 (100|100)

ASV36 *Phaeocystis globosa*	KC900889.1, MT471334.1 (100 | 100)
ASV62 *Plagiogrammopsis vanheurckii*	NC_037998.1, NC_037997.1 (98.42-98.43|100) – also had high similarity to *Guinardia striata*
ASV41 *Plagioselmis* sp.	AB164406.1 (98.95|100)
ASV72 *Pseudopedinella elastica*	NC_044408.1 (99.21|100)
ASV8 *Pyramimonas disomata*	FN563101.1* (100 | 100)
ASV39 *Pyramimonas disomata*	FN563101.1* (100|100)
ASV63 *Skeletonema pseudocostatum*	MK372941.1 (100|100)
ASV64 *Skeletonema pseudocostatum*	MK372941.1 (99.74|100)
ASV28 *Stephanopyxis nipponica*	LN735463.3, AJ536465.1 (100 | 100) – was also similar to *Stephanopyxis turris,* however, species name not given in sequence description for LN735463.3
ASV65 *Stephanopyxis* sp.	FJ002176.1, AJ536465.1 (100|100)
ASV12 *Synechococcus* sp.	CP047959.1 (100 | 100)
ASV13 *Synechococcus* sp.	JX530065.1, CP011941.1 (99.71-100 | 100)
ASV14 *Synechococcus* sp.	MH358353.1, MT994359.1 (100 | 100)
ASV44 *Synechococcus* sp.	JX529910.1, JX477000.1 (99.47|100)
ASV43 *Synechococcus* sp.	CP047959.1, CP047942.1 (100|100)
ASV45 *Synechococcus* sp.	LN847356.1 (100)
ASV10 *Teleaulax amphioxeia*	KP899713.1* (100 | 100)
ASV11 *Teleaulax amphioxeia*	KP899713.1* (99.43 | 100)
ASV42 *Teleaulax amphioxeia*	KP899713.1*, EU123322.1 (100|100)
ASV9 *Tetraselmis convolutae*	LN735267.2 (98.29 | 100)
ASV40 *Tetraselmis* sp.	HE610165.1, KU167097.1 (93.63-96.25|98) – matches more closely to *Tetraselmis cordiformis*, however, alignments for these accessions only have 98% query coverage and <97% PI.
bASV66 *Thalassiosira oceanica*	GU323224.1 (98.95|100)
ASV67 *Thalassiosira pseudonana*	EF067921.1 (99.74|100)
ASV30 *Thalassiosira* sp.	KT956318.1, LN735461.3, LN735460.3 (100 | 100)
ASV31 *Thalassiosira* sp.	MH011825.1, KT956312.1 (100 | 100)
bASV29 Thalassiosirales sp.	MK372941.1, GU323224.1 (100 | 100)
ASV68 Thalassiosirales sp.	Now possibly *Coscinodiscus wailesii** (new sequence Apr-2021 MW561224.1 (98.42|100))

Table S3. Additional ASVs dominant on the Scotian Shelf that were also manually identified. Information is reported using the same approach described in the caption of Table S2 (see above).

Taxon	Accessions (PI % | Query Coverage %)
cASV1 *Picochlorum* sp.	MN647759.1 (98.69 | 100)
cASV2 *Trichodesmium thiebautii*	AF091321.1 (99.74 | 100); MT478931.1 (100 | 100)
cASV3 *Synechococcus* sp.	JX477000.1 (100 | 100)
cASV4 *Synechococcus* sp.	CP047954.1 (100 | 100); CP047949.1 (100 | 100)
cASV5 *Prochlorococcus* sp.	CP007754.1 (100 | 100)
cASV6 *Prochlorococcus* sp.	CP018346.1, CP018345.1, CP018344.1 (all 100 | 100)
cASV7 *Nostocales* sp.	AB491868.1 (95.2|98); MT488223.1 (93.39|99); AM230674.1 (93.39|99)
cASV8 *Cyanobacterium* sp.	None
cASV9 *Thalassiosira pseudonana*	FJ002218.1, EF067921.1 (both 99.21|100)
cASV11 *Leyanella arenaria*	FJ002242.1 (99.47 | 100)
cASV12 *Leptocylindrus danicus*	KC509524.1 (99.74 | 100)
cASV13 *Fragilariopsis* sp.	NC_045244.1, LR812620.1, FJ002238.1 (all 97.64 | 100)
cASV14 *Bacteriastrum hyalinum*	FJ002166.1 (98.42 | 100)
cASV15 *Bacillariophyta* sp.	None
cASV16 *Bacillariophycidae* sp.	KM218905.1, FJ002217.1, FJ002224.1, GU591328.1 (all 98.43 | 100)
cASV17 *Bacillaria paxillifer*	AJ536452.1 (98.42 | 100)
cASV18 *Eutreptiella pomquetensis*	KY706202.1 (99.74 | 100)
cASV19 *Emiliania huxleyi*	X82156.1 (100 | 100); JN022705.1 (99.74 | 100); AY741371.1 (99.74 | 100)
cASV20 *Braarudosphaera bigelowii*	AB847986.2 (100 | 100)
cASV21 *Pelagomonas* sp.	JX297813.1 (100 | 100)
cASV22 *Pelagomonas* sp.	JX297813.1 (99.74 | 100)
cASV23 *Pelagomonas* sp.	JX297813.1 (98.68 | 100)
